# NADH elevation during chronic hypoxia leads to VHL-mediated HIF-1α degradation via SIRT1 inhibition

**DOI:** 10.1186/s13578-023-01130-3

**Published:** 2023-09-30

**Authors:** Hyun-Yoo Joo, Jin Kyu Jung, Mi-Yeon Kim, Seon Rang Woo, Jae Min Jeong, Eun-Ran Park, Yong-Min Kim, Joong-Jean Park, Joon Kim, Miyong Yun, Hyun-Jin Shin, Kee-Ho Lee

**Affiliations:** 1 Division of Radiation Biomedical Research, Korea Institute of Radiological & Medical Sciences, Seoul, Korea; 2 Department of Physiology, College of Medicine, Korea University, Seoul, Korea; 3 Laboratory of Toxicology, College of Veterinary Medicine, Seoul National University, Seoul, Korea; 4 Lab. of Biochemistry, School of Life Sciences & Biotechnology, Korea University, Seoul, Korea; 5 Department of Bioindustry and Bioresource Engineering, College of Life Sciences, Sejong University, Seoul, Korea; 6 Neuro-Oncology Branch, The Center for Cancer Research, National Cancer Institute, National Institutes of Health, Bethesda, MD USA; 7 Department of Otolaryngology-Head and Neck Surgery, Kyung Hee University School of Medicine, Hyung Hee University Medical Center, Seoul, Republic of Korea

**Keywords:** Chronic hypoxia, NADH elevation, HIF-1α degradation, SIRT1, VHL, Invasion, Angiogenesis

## Abstract

**Background:**

Under conditions of hypoxia, cancer cells with hypoxia inducible factor-1α (HIF-1α) from heterogeneous tumor cells show greater aggression and progression in an effort to compensate for harsh environmental conditions. Extensive study on the stability of HIF-1α under conditions of acute hypoxia in cancer progression has been conducted, however, understanding of its involvement during the chronic phase is limited.

**Methods:**

In this study, we investigated the effect of SIRT1 on HIF1 stability in a typical chronic hypoxic conditon that maintains cells for 24 h under hypoxia using Western blotting, co-IP, measurement of intracellular NAD + and NADH levels, semi-quantitative RT-PCR analysis, invasion assay, gene knockdown.

**Results:**

Here we demonstrated that the high concentration of pyruvate in the medium, which can be easily overlooked, has an effect on the stability of HIF-1α. We also demonstrated that NADH functions as a signal for conveyance of HIF-1α degradation via the SIRT1 and VHL signaling pathway under conditions of chronic hypoxia, which in turn leads to attenuation of hypoxically strengthened invasion and angiogenic activities. A steep increase in the level of NADH occurs during chronic hypoxia, leading to upregulation of acetylation and degradation of HIF-1α via inactivation of SIRT1. Of particular interest, p300-mediated acetylation at lysine 709 of HIF-1α is recogonized by VHL, which leads to degradation of HIF-1α via ubiquitin/proteasome machinary under conditions of chronic hypoxia. In addition, we demonstrated that NADH-elevation-induced acetylation and subsequent degradation of HIF-1α was independent of proline hydroxylation.

**Conclusions:**

Our findings suggest a critical role of SIRT1 as a metabolic sensor in coordination of hypoxic status via regulation of HIF-1α stability. These results also demonstrate the involvement of VHL in degradation of HIF-1α through recognition of PHD-mediated hydroxylation in normoxia and p300-mediated HIF-1α acetylation in hypoxia.

**Supplementary Information:**

The online version contains supplementary material available at 10.1186/s13578-023-01130-3.

## Background

In most cases, hypoxia occurs in chronically and acutely developed solid human cancers, due to the reduction in oxygenation diffusion with increased distance from the vasculature, and decreased capacity for oxygen transport of blood leading to development of poor clinical symptoms and treatment outcomes [1–6]. Chronic hypoxia occurs in condition with prolonged hypoxic stress due to excessive proliferation of cancer with high density leading to oxygen deprivation by extended period, and acute hypoxia occurs within short time frames, ranging from minutes to a few hours. It arises due to the sudden closure of blood vessels caused by the mass of cancer or irregular blood cell flow [8]. Obvious differences in the biological and therapeutic consequences as well as pathophysiology beween acute and chronic hypoxia have been reported [7, 8]. Disassembly of the replisome induced by chronic hypoxia exposure prevents replication resumption even after reoxygenation [9, 10]. According to the results of in vitro analysis for assessment of radiationsensitivity, cells under conditions of chronic hypoxia are consistently more sensitive to radiation therapy than those under acute conditions. Several reports showing the results of clinical analysis have indicated that acute hypoxia results in progression to a more aggressive phenotype [11–14]. Despite these differences in biological and clinical consequences between acute and chronic hypoxia, no known molecular mechanisms that can explain the reason has been discovered thus far.

Acqusition of aggressive phenotypes observed in hypoxic tumors is mainly attributed to hypoxia-inducible factor-1α (HIF-1α), which plays a central role in management of hypoxic stress: HIF-1α transcriptionally provides hundreds of target proteins for adaptation of cancer cells [15]. Under conditions of normoxia, degradation of HIF-1α occurs via hydroxylation by proline hydroxylase (PHD) and subsequent recruitment of von-Hippel Lindau (VHL) tumor suppressor with E3 ligase activity [16, 17]. Under conditions of hypoxia, detachment of HIF-1α from VHL occurs immediately via inactivation of PHD, resulting in rapid stabilization [18, 19]. Acute and chronic hypoxic conditions with distinct phenotypic differences are majorly regulated by variations in the functioning of the HIF-1α and HIF-2α transcription factors, which respond to acute conditions and adjust to chronic conditions, respectively. HIF-1α is degraded upon prolonged hypoxic stress while HIF-2α is stabilized [20, 21]. However, HIF-1α during periods of prolonged hypoxia can sometimes be maintained without destruction [22]. In the last two decades since its discovery, a variety of upstream regulators of HIF-1α protein during the acute phase of hypoxia have been discovered and the stabilizing mechanisms have been defined [23]. In contrast, understanding of the HIF-1α degradation pathway under conditions of chronic hypoxia remains limited.

Extensive research focusing on regulation of HIF-1α activity by members of the SIRT family has recently been conducted, and its mechanisms for stabilization and transcriptional control have been defined. SIRT1 [24, 25], SIRT2 [26], SIRT3 [27, 28], and SIRT6 [29], from the sirtuin family, have a direct effect on the stability of HIF-1α. In particular, SIRT1 is known to control the activity of HIF-1α under hypoxic condtions in various cancers. However, findings from the studies reporting on regulation of HIF-1α activity by SIRT1 are debatable. The earlier study examining transcriptional control of HIF-2α proposed that SIRT1 has no effect on HIF-1α [30]. Based on the result showing that SIRT1 has an impact on both HIF-2α and HIF-1α, this finding is contradictory: SIRT1 has negative control of HIF-1α transcription [25, 31]. However, the conclusion does not support the result showing that activity of HIF-1α is positively controlled by SIRT1 through stabilization of the protein [24, 32]. These controversial results are based on study conducted under conditions where acute hypoxia cannot be distinguished from chronic hypoxia, despite distinctive differences in biological, pathophysiological, and medical consequences as well as the level of HIF-1α protein between the two types of hypoxia.

In the current study, we attempted to determine whether there is also an association of SIRT1 with HIF-1α during chronic hypoxia; previous studies have reported on assessment performed during the acute phase of hypoxia [25]. We also examined the effects of high levels of nutrients, particularly nutrients such as pyruvate in cell culture media, on the stability of HIF-1α during chronic hypoxia. Of particular interest, we found that elevation of NADH, a major energy metabolite, is involved in regulation of HIF-1α stability via the SIRT1/VHL pathway during chronic hypoxia. Our current conclusion provides a resolution for several issues regarding chronic hypoxia: the reason why cancer cells lacking *VHL* are not capable of degrading HIF-1α during prolonged periods of hypoxia and why cancer cells show less agression under conditions of chronic hypoxia- compared with those of acute-hypoxia, and the mode of action of metabolites in chronic degradation of HIF-1α.

## Results

### Pyruvate in cell culture media directly affects HIF-1α stability during chronic hypoxia

Hypoxic tumor cells under physiological conditons have a limited supply of nutrients as well as oxygen, which has been overlooked in most currently ongoing in vitro experiments. Although careful consideration of glucose and pyruvate, which can directly induce accumulation of HIF-1α [33, 34], is required in conduct of hypoxic experiments using cancer cells, many previous reports regarding the stability of HIF-1α have been conducted using media containing high levels of nutrients such as DMEM instead of MEM without pyruvate and glucose. Cancer cells treated with DMEM or MEM were used to determine the effect of DMEM containing high levels of glucose and pyruvate on the stability of HIF-1α under conditions of hypoxia. As expected, accumulation of HIF-1α in HT1080 and HeLa was observed at 9 h under both DMEM and MEM conditions (Fig. S1A). However, upon prolonged hypoxic stress (24 and 48 h), the expression level of HIF-1α in cultured cells was sustained or increased at 48 h in DMEM, while decreases in MEM were observed (Fig. S1A). Acute hypoxia progresses in minutes to several hours, and degradation HIF-1α under prolonged hypoxic condition is a typical phenomenon of chromic hypoxia [20, 21]. Therefore, we will classify this prolonged hypoxic stress lasting more than 24 h as chronic hypoxia, aligning with the definition employed by other studies [8].

Next, pyruvate was added to MEM in order to examine the role of media nutrients in stability of HIF-1α during chronic hypoxia. The results showed that addition of pyruvate to MEM inhibited the reduction of HIF-1α during chronic hypoxia, similar to that observed in DMEM (Fig. S1B). These data strongly suggest that pyruvate can affect the stability of HIF-1α protein during chronic hypoxia.

### NADH functions as a signal triggering chronic HIF-1α decay

**Fig. 1:**
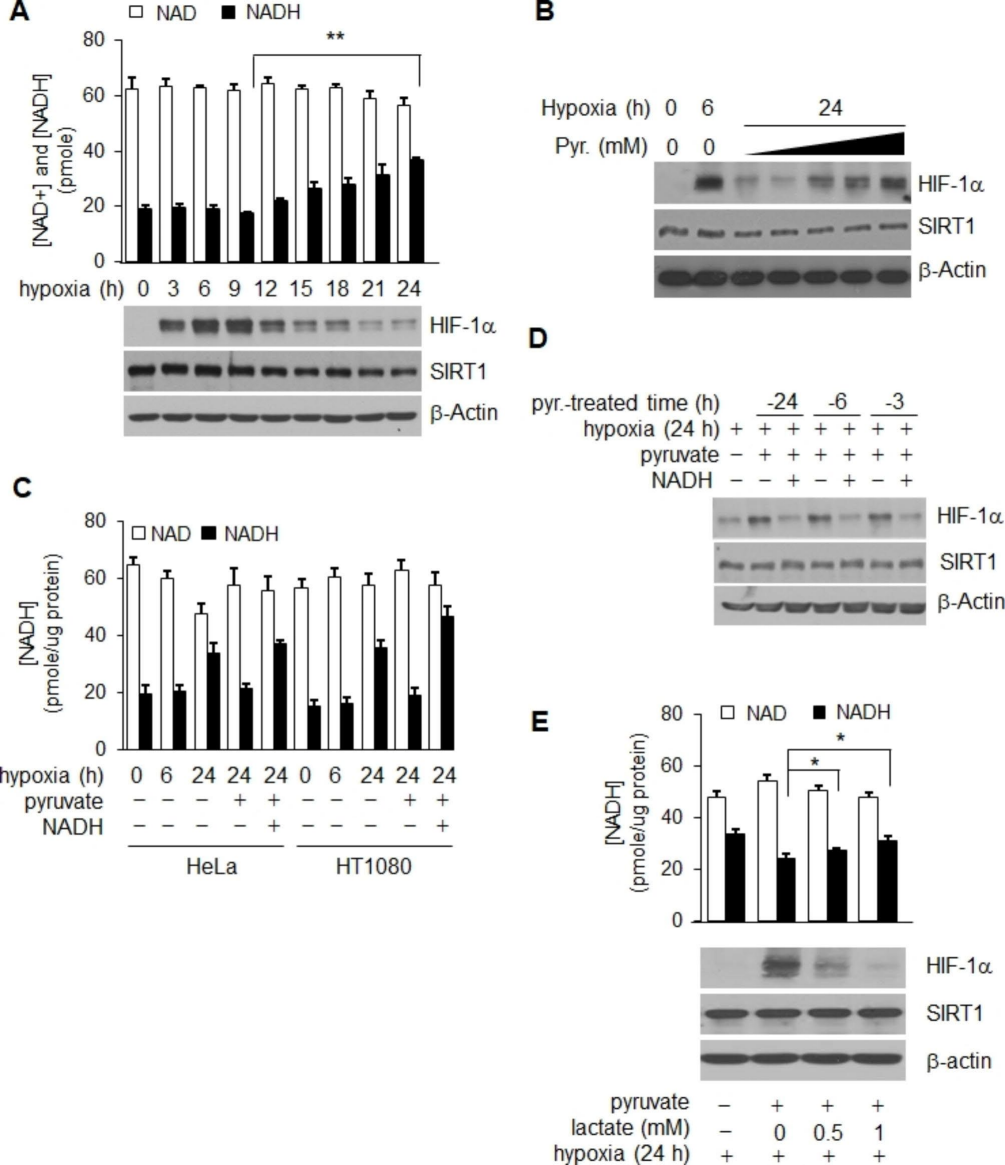
NADH controls HIF-1α decay via involvement of SIRT1 during chronic hypoxia. (**A**) The timecourse of HIF-1α protein levels and intracellular NAD^+^ and NADH concentrations was determined in HeLa cells over 24 h of hypoxia. (**B ~ E**) Both the extent of NADH oxidation and the HIF-1α maintenance or recovery levels induced by pyruvate were determined by measurement of NAD^+^ and NADH concentrations, and HIF-1α protein, all under hypoxic conditions. Pyruvate (**B**) or NADH (**C,D**) or lactate (**E**) was added to confirm the existence of NADH-sensitive HIF-1α rescue. Statistical significance (*p*-value) was determined using the ANOVA-t-test. * : *p* < 0.05, ** : *p* < 0.01. NADH and pyruvate were added at a concentration of 1 mM

We initially compared the level of HIF-1α protein with those of intracellular NAD^+^ and NADH in HeLa cells growing under hypoxic and in MEM media conditions in order to examine the impact of the NAD^+^ redox potential on the stability of HIF-1α during hypoxia. The results of a 24 h experiment conducted under hypoxic conditions showed that the level of NADH was significantly increased (p = 0.001), as previously reported [35–37], and the increase showed a close association with decay of HIF-1α protein (Fig. 1A). In contrast, no substantial change in the concentration of NAD^+^ was observed (p = 0.071, Fig. 1A). To further examine the association between NADH and the stability of HIF-1α during hypoxia, pyruvate, an NADH converter [36, 38], was added to culture media under hypoxic conditions. When pyruvate was present throughout the entire 24 h of hypoxic exposure, the decreased level of HIF-1α was almost recovered, similar to that observed with 6 h and was dependent on the concentration of pyruvate (Fig. 1B). The intracellular concentration of NADH was elevated by addition of NADH to the same set of pyruvate-treated hypoxic cultures in order to determine whether the effect of pyruvate on the level of HIF-1α was attributable to an alteration in the concentration of NADH (Fig. 1C). As shown in Fig. 1D, the level of HIF-1α was decreased by the increased level of NADH without change in the SIRT1 protein level. In addition, the pyruvate-mediated recovery of HIF-1α levels was reduced by addition of lactate accompanied by an increase in the level of NADH (*p* < 0.05, Fig. 1F). These findings indicate that the hypoxic level of HIF-1α is sensitive to change in the concentration of intracellular NADH, and that elevation of NADH during prolonged chronic hypoxia induces decay of HIF-1α. Upon prolonged hypoxic conditions, the expression of SIRT1 exhibited a slight decrease, and its activity notably diminished, due to the increased levels of NADH (Fig. 1A). Hence, it can be inferred that the degradation in HIF-1α during chronic hypoxia is primarily attributed to the diminished activity of SIRT1.

### NADH-mediated HIF-1α stablility is correlated with SIRT1 activity in chronic hypoxia

**Fig. 2:**
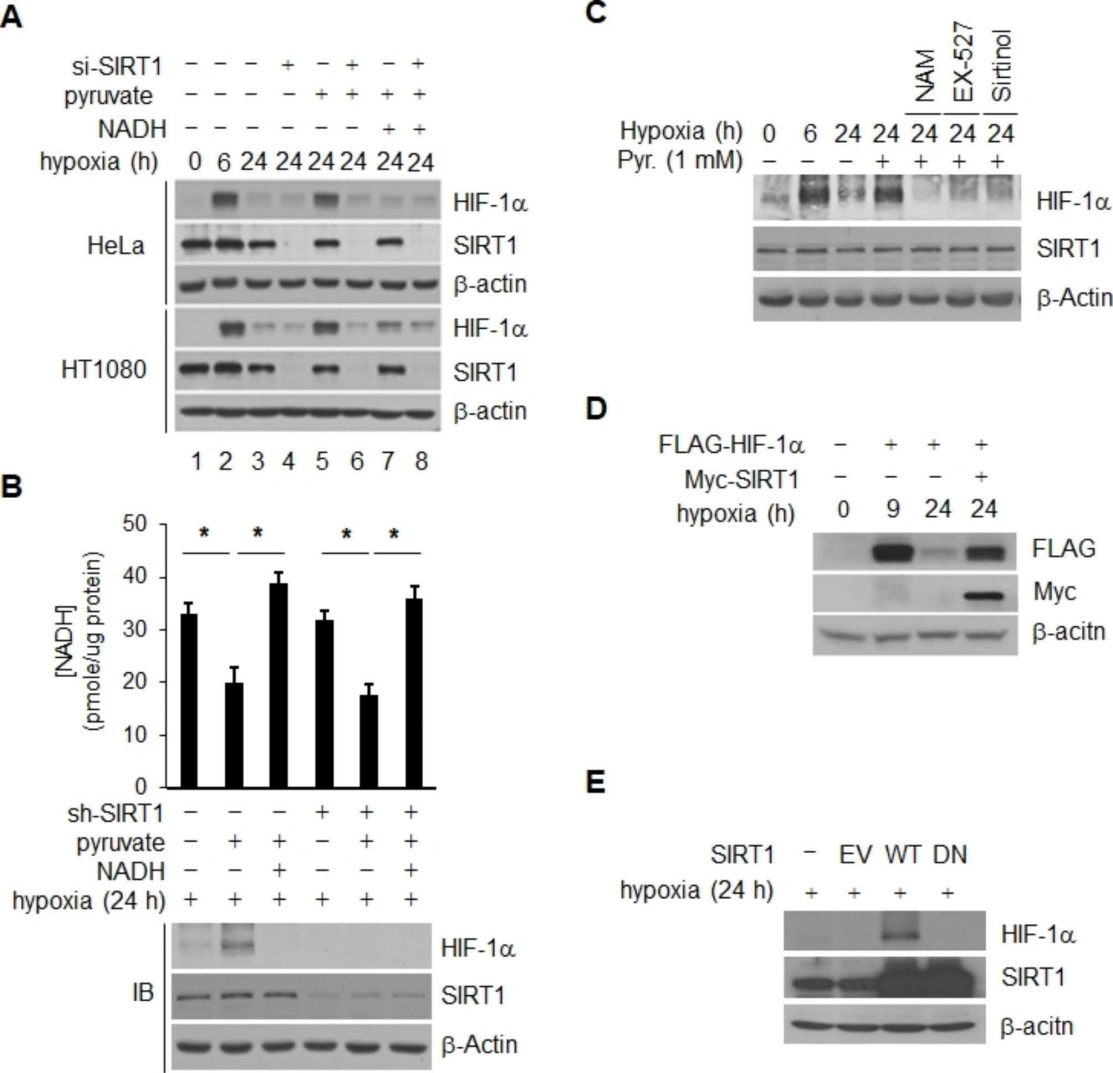
SIRT1-specific regulatd HIF-1α stability under hypoxic conditions. (**A** and **B**) Both the extent of NADH oxidation and the HIF-1α maintenance or recovery levels induced by SIRT1 were determined by measurement of NADH concentrations and HIF-1α protein in the presence of SIRT1-siRNA **(A)** or SIRT1- shRNA **(B)**. **(C)** Association of SIRT1 activity with pyruvate-mediated recovery of HIF-1α was also validated in HeLa cells incubated with or without the SIRT1 inhibitors NAM (20 mM), EX-527 (1 µM), or sirtinol (25 µM); these inhibitors were present from commencement of hypoxia to 24 h, and 1 mM pyruvate was added 6 h before harvesting. Chronic decay of HIF-1α was monitored in HeLa cells cotransfected with *Flag*-tagged *HIF-1α* and either *Myc*-tagged wild type-*SIRT1* or empty vector (-) **(D)**; or in HeLa cells expressing wild-type *SIRT1* (WT), dominant-negative *SIRT1* (DN), or empty vector (V) **(E)**. Statistical significance (*p*-value) was determined using the ANOVA-t-test. * : *p* < 0.05

Activity of SIRT1 is inhibited by intracellular NADH [39, 40] and inactivation of SIRT1 leads to inhibition of early accumulation of hypoxic HIF-1α [24, 32], suggesting that chronic decay of HIF-1α may result from inactivation of SIRT1 through elevation of NADH. Therefore, we examined the effect of SIRT1 on chronic decay of HIF-1α in the same set of culture. Under the condition of SIRT1 inhibition with transfection of *SIRT1*-siRNA or addition of NADH, pyruvate did not prevent decay of HIF-1α in HeLa and HT1080 cells (Fig. 2A). Elimination of the pyruvate effect was also obtained using three different siRNAs targeting different coding sequences on *SIRT1* (Fig. S1C). S1RT1 depleted clones were constructed by incorporation of *sh-SIRT1* in order to examine the role of SIRT1 in pyruvate mediated recovery of HIF-1α in chronic hypoxia. Indeed, the pyruvate effect on HIF-1α was not observed in SIRT1-depleted clonal populations, despite the lower level of NADH (Fig. 2B and Fig. S1D). In addition, the level of redox couple NAD^+^ or NADH was not altered by depletion of SIRT1 (Fig. 2B). Application of SIRT1 inhibitors NAM [41], EX-527 [42], and Sirtinol [43] also resulted in elimination of the pyruvate effect (Fig. 2C). These findings demonstrate that pyruvate-mediated prevention of HIF-1α decay does not occur under the condition of SIRT1 depletion or inactivation. To further confirm these findings, we examined the question of whether overexpression of SIRT1 can prevent chronic decay of HIF-1α. As expected, chronic decay of HIF-1α was inhibited by transfection of *Myc*-tagged wt-*SIRT1* in HeLa cells (Fig. 2D). The inhibitory effect on decay of HIF-1α was not observed in cells transfected with a dominant negative *SIRT1* (*SIRT1*/H363Y) [44] ( Fig. 2E). MG132, a potent inhibitor of 26 S proteosomal proteolysis, was added to hypoxic cells in order to determine whether our current observation, NADH-sensitive SIRT1 regulation of chronic HIF-1α decay, is also mediated by proteosomal degradation. According to our findings, treatment with MG132 increased the level of HIF-1α protein that was decreased during prolonged hypoxia (Fig. S2A) and this stabilization remained unaffected by the depletion of SIRT1 and/or pyruvate treatment, indicating that the regulation of HIF-1α by SIRT1 and pyruvate is mediated through the ubiquitin-proteasome pathway (Fig. S2B). In addition, the level of *HIF-1α* mRNA was constantly maintained during prolonged hypoxia and was not affected by a change in the concentrations of NADH and pyruvate (Fig. S2C and S2D). In addition, depletion of SIRT1 had no effect on the levels of *HIF-1α* mRNA in cells under these conditions (Fig. S2D). These data indicate that NADH-mediated stability of HIF1 is intricately regulated by the activity of SIRT1 under conditions of hypoxia.

### NAD^+^ produced via the actions of the AMPK and NAMPT pathways triggers HIF-1α stabilization dependent on SIRT1

**Fig. 3:**
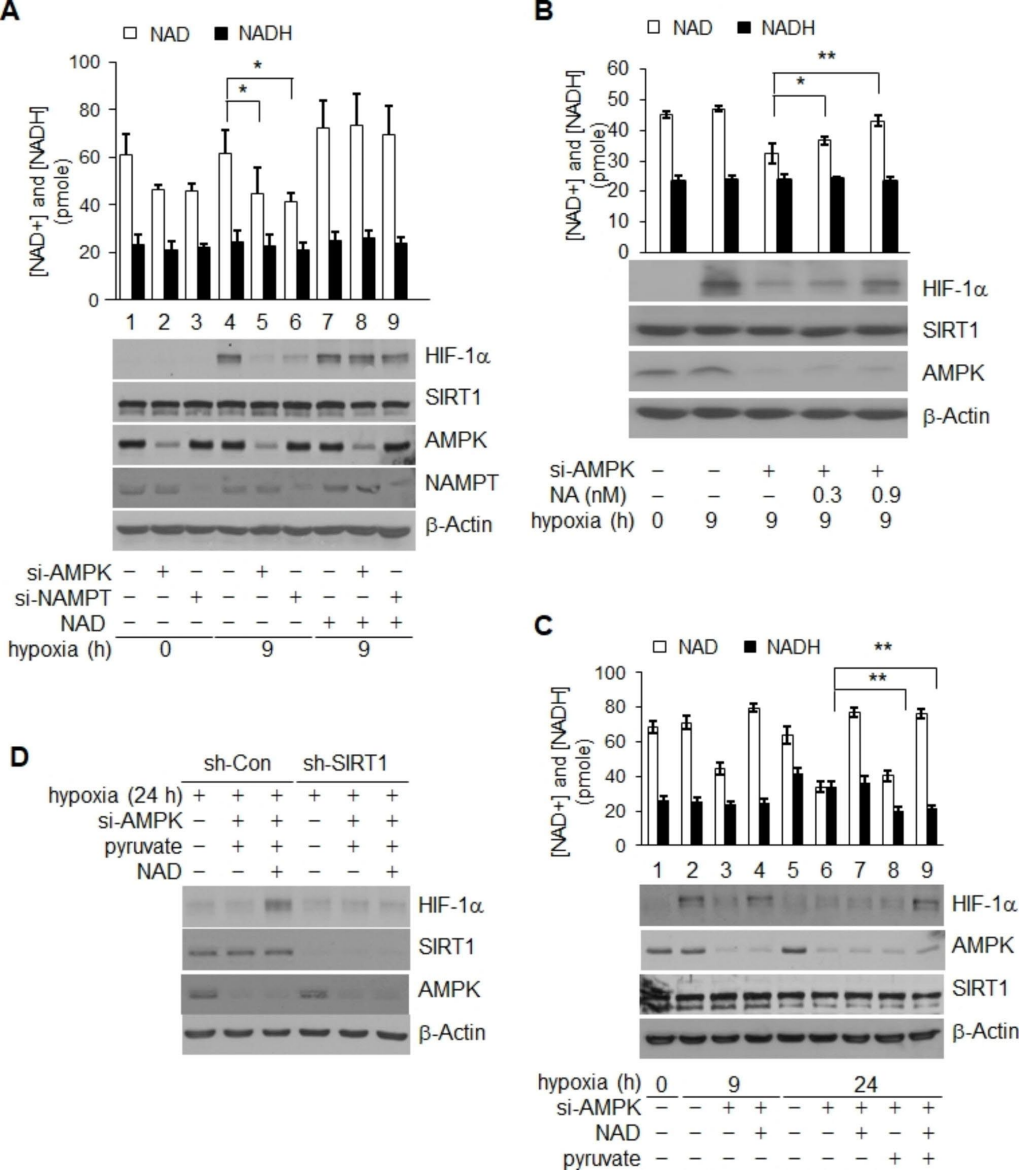
NAD^+^ synthesized via AMPK and NAMPT pathways stabilizes HIF-1α during hypoxia. (**A** and **B**) NAD^+^-sensitive HIF-1α accumulation. HIF-1α protein levels were determined under NAD^+^-reduced and -reconstituted conditions after hypoxic exposure for 9 h; NAD^+^ limitation was achieved via use of NAMPT- or AMPK-siRNA whereas NAD^+^ reconstitution involved external addition of 1 mM NAD^+^ (**A**) or nicotinic acid (NA) (**B**). (**C**) NAD^+^/NADH-sensitive recovery in HIF-1α levels. Pyruvate or NAD-mediated recovery in HIF-1α levels was measured under NAD^+^-reduced and NAD^+^-reduced-plus-NADH-upregulated conditions, upon hypoxic exposure for 9 and 24 h, respectively; tests employed AMPK-siRNA treatment. (**D**) The need for SIRT1 expression in terms of NAD^+^/NADH-sensitive HIF-1α recovery was evaluated in HeLa cells in which SIRT1 was or was not stably depleted, using *SIRT1*- or control-shRNA, respectively. Statistical significance (*p*-value) was determined using the ANOVA-t-test. * : *p* < 0.05, ** : *p* < 0.01

The finding that chronic decay of HIF-1α is promoted by elevation of NADH and consequent inactivation of SIRT1, along with the fact that activity of SIRT1 is dependent on NAD+, raised the question of whether the level of NAD + was also important for SIRT1-mediated stabilization of HIF-1α. To further examine this question, the level of cellular NAD^+^ was decreased by inhibition of either the AMPK or NAMPT pathway, which are both critical in biosynthesis of NAD^+^ [45, 46]. Inhibition of either pathway resulted in a significant reduction of early hypoxic accumulation of HIF-1α, accompanied by declines in the concentration of NAD^+^ (34.5% and 37.8%, respectively) (Fig. 3A, lanes 4, 5 and 4, 6). This impaired accumulation of HIF-1α through inhibition of either the AMPK or NAMPT pathway was reversed by addition of NAD^+^ for supplementation of intracellular NAD^+^ (p < 0.05) (Fig. 3A, lanes 6, 8 and 6, 9, and Fig. S3A). In addtion, this finding was confirmed by treatment with nicotinic acid, which is metabolized to NAD^+^ through a salvage pathway (p < 0.05) (Fig. 3B) [46, 47]. However, addition of NAD^+^ under normixic condition or without depletion of AMPK or NAMPT under hypoxic conditions, did not induce further accumulation of HIF-1α (Fig. S3B and Fig. 3A, lanes 4, 7). These results demonstrated that the normoxic level of NAD^*+*^ is adequate for accumulation of HIF-1α under conditions of acute hypoxia. Degradation of HIF-1α was also prevented by MG132 upon depletion of AMPK or NAMPT (Fig. S4A and B).

To further develop this idea, we next created chronic hypoxic conditions, via inhibition of the AMPK pathway, resulting in attainment of redox couple NAD^+^ and NADH levels greatly below and in excess of those characteristic of normoxia, respectively (Fig. 3C). Under these conditions, neither an increase in the level of NAD^+^ upon exogenous supplementation (lanes 6, 7), nor a decrease in the level of surplus NADH caused by addition of pyruvate (lanes 6, 8) resulted in rescue of HIF-1α accumulation (Fig. 3C). However, addition of both pyruvate and NAD^+^ resulted in complete rescue of HIF-1α recovery via supplementation with NAD^+^ (lanes 6, 9) and reduction of NADH (lanes 6, 9) (Fig. 3C); this was dependent on expression of SIRT1, since recovery of HIF-1α was eliminated by depletion of SIRT1 (Fig. 3D). Thus, the initial extent of HIF-1α stabilization under hypoxic conditions is determined by the level of intracellular NAD^+^, but only in the presence of SIRT1.

### NADH-mediated SIRT1 inactivation increases HIF-1α acetylation in chronic hypoxia

**Fig. 4:**
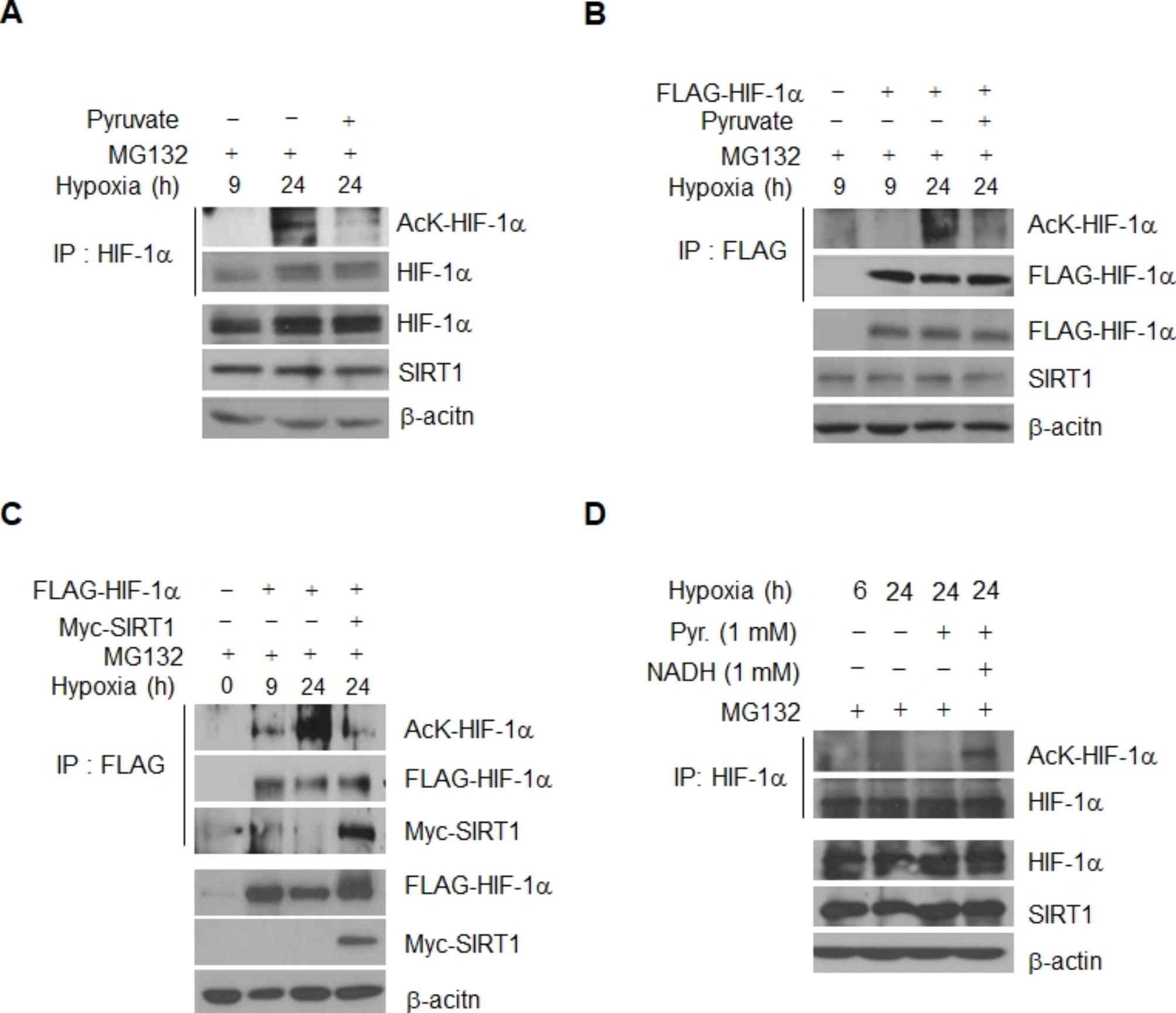
HIF-1α acetylation is increased during chronic hypoxia. **(A** ~ **D)** HIF-1α acetylation was determined in immunoprecipitated HIF-1α preparations, followed by probing with anti-acetyl-K antibody, in HeLa cells exposed to hypoxia for 9 and 24 h, under condition of MG132 treatment. HIF-1α immunoprecipitates were prepared with anti-HIF-1α or anti-Flag-antibodies from the cells accumulating HIF-1α endogenously (**A**) or exogenously through transfection with *Flag*-tagged *HIF-1α* (**B** and **C**). The effect of chronic hypoxia condition **(A)**, pyruvate (**B**), and *Myc*-tagged *SIRT1* transfection (**C**) on the acetylation was monitored. (**D**) Pyruvate/NADH-sensitive recovery in HIF-1α acetylation. HIF-1α acetylation was determined in the presence of pyruvate or NADH under chronic hypoxia condition

Two earlier reports, including ours [24, 32], showed that depletion or inactivation of SIRT1 leads to an increase in acetylation of HIF-1α protein, and suggested that activation of SIRT1 and deacetylation of the protein is required for hypoxic stabilization of HIF-1α. These previously reported findings and our current data suggest that chronically elevated NADH promotes degradation of HIF-1α via inactivation of SIRT1. Therefore, we then examined the question of whether chronic degradation of HIF-1α occurs via the protein acetylation induced by inactivation of SIRT1. Substantially increased acetylation of HIF-1α was observed on both endogenous (Fig. 4A) and exogenously overexpressed protein 24 h after hypoxic exposure (Fig. 4B), compared to that observed at 9 h (Fig. 4A and B). The chronically increased acetylation was reduced by overexpression of pyruvate (Fig. 4A and B) or SIRT1 (Fig. 4C). Addition of NADH resulted in a consistent reversal of the effect of pyruvate, resulting in the re-elevation of HIF-1α acetylation even after addition of pyruvate (Fig. 4D). The data imply that acetylated HIF-1α is regulated by the NADH/SIRT1 signal pathway during the chronic phase.

### Acetylation of HIF-1α lysine 709 is coordinated by SIRT1 and acetyltransferase p300

To examine regulation by HIF-1α acetylation, we modulated the expression of acetylatransferases, which were previously reported as regulators of HIF-1α. A recent study reported that ARD-1 has an important role in regulation of HIF-1α stability via acetylation [48]. ARD-1 and SIRT1 were induced in cells in order to determine the relationship of ARD-1 mediated acetylation of HIF-1α with SIRT1. Increased SIRT1 did not result in a change in the level of HIF-1α acetylation by ARD-1 (Fig. S5A). In addition, induction of the HIF-1α K532R (ARD-1 mediated acetylation site) mutant led to a decrease of SIRT1-depleted condtions, similar to that observed with wildtype, but neither K709R nor K709R/K532R (Fig. S5B), indicating that ARD-1 is not involved in stabilization of HIF-1α by SIRT1.

**Fig. 5:**
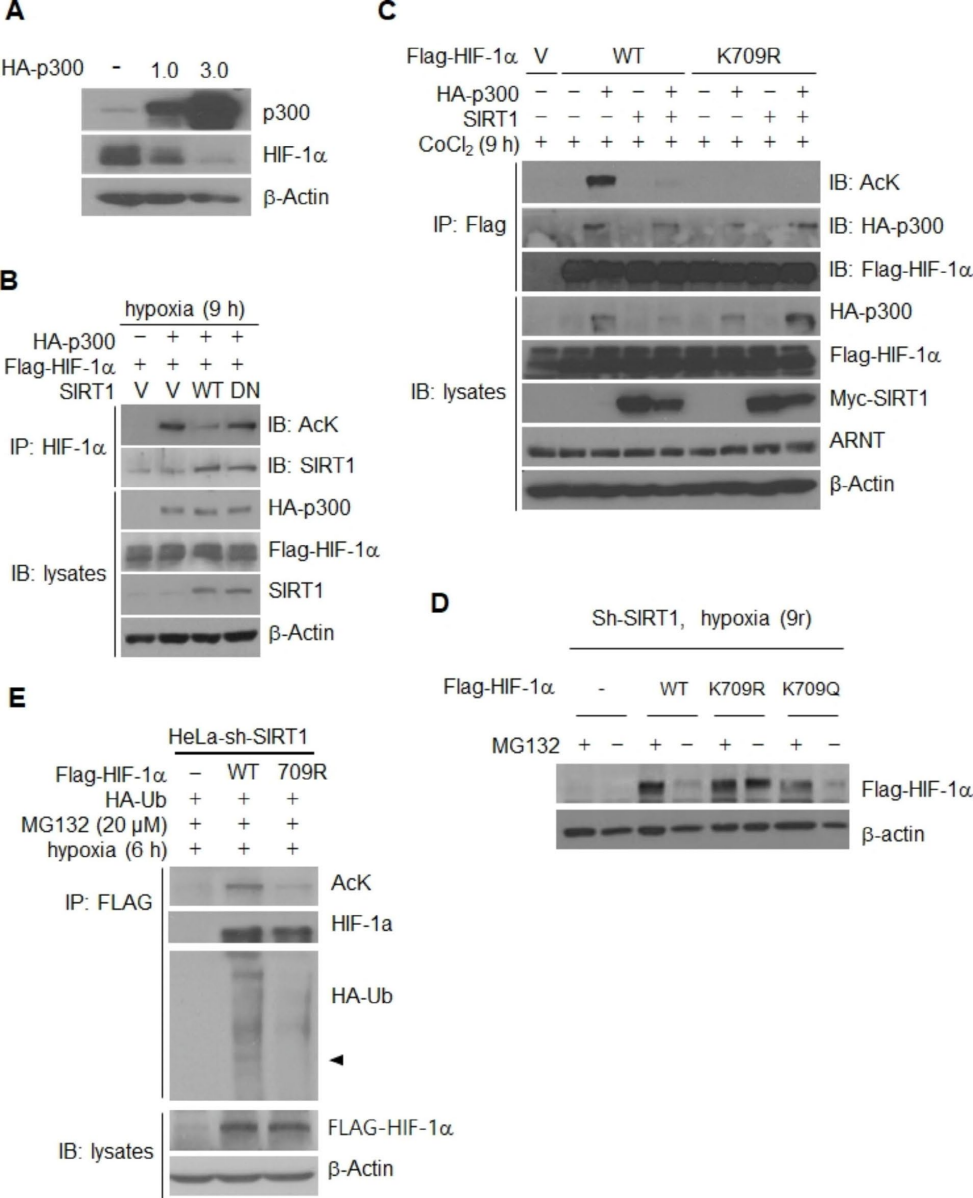
SIRT1 deacetylates Lys709 of HIF-1α to facilitate protein stabilization, even under conditions of protein dehydroxylation. **(A)** Overexpression of p300 regulated HIF-1a stability. HIF-1α protein levels were determined under hypoxic exposure for 9 h after overexpression of p300. **(B)** Deacetylation of *HIF-1α* by SIRT1. 293T cells were transiently transfected with Flag-tagged *HIF-1α* and HA-tagged p300 together with wt-SIRT1 (WT) or SIRT1/H363Y (DN). *HIF-1α* acetylation and HIF-1α-SIRT1 interaction in immunoprecipitated *HIF-1α* were assessed by probing with antibodies to acetylated lysine (AcK), and SIRT1, respectively. **(C)** 293T cells were transiently cotransfected with 2 µg of each of Flag-tagged *HIF-1α*, K709R or empty vector, and 2 µg of each of HA-tagged p300 and/or Myc-tagged SIRT1. **(D)** The stability of K709Q was compared to that of K709R and wt-HIF-1α in SIRT1-depleted HeLa cell clone either treated or not with MG132. **(E)** Acetylation and ubiquitination of immunoprecipitated WT-*HIF-1α* and K709R were assessed in in SIRT1-depleted HeLa cell clone

Next, we had a concern that p300 is a well documented large acetyltransferase affecting the stability or activity of many types of proteins. Stability of HIF-1α is regulated by p300 via acetylation on lysine 709, although some evidence is controversial [49]. In order to determine whether stability of HIF-1α is regulated by p300 in our system, p300 was overexpressed in cancer cells. Figure 5 A shows that expression of HIF-1α is downregulated by p300. In addition, p300-mediated acetylation of HIF-1α was decreased by induction of SIRT1, while SIRT1 DN (dominant negative) had no effect (Fig. 5B). Next, deletion mutants were constructed in order to determine the site of acetylation of HIF-1α by p300, (Fig. S6A and B) and post-translational modification analysis was performed using Maldi tof for evaluation of HIF-1α (Fig. S6C). The results of these analyses indicated that lysine 709 of HIF-1α is acetylated by p300 and deacetylated by SIRT1 (Fig. S6B, C and Fig. 5C). In addition, under the condition of SIRT1 depletion in hypoxia, the levels of wild type and HIF-1α K709Q mutant (acetylation mimic) protein were decreased, while that of K709R was not (Fig. 5D). In an analysis to determine whether this acetylation site has an effect on the protein stability of HIF-1α, we observed that both acetylation and ubiquitination of the 709R mutant was significantly lower than those for wild type HIF-1α (Fig. 5E), suggesting the importance of acetylation on HIF-1α K709 in regulation of SIRT1 dependent stability of HIF-1α under conditions of hypoxia. In some experimental condition, HIF-1α K709R did not show a complete disappearance of acetylation (Fig, 5E), which indicates the presence of other acetylation sites on HIF-1α. However, HIF-1α K709R was not degraded in SIRT1 depletion condition. The stabilized protein level was similar to that observed with MG132 treatment, the condition in which all regulatory mechanisms regarding ubiquitin-proteasome degradation pathways are rendered inactive (Fig. 5D). Additionally, HIF-1α K709R exhibited nearly complete inhibition of ubiquitin ligation from SIRT1 depletion (Fig. 5E). Therefore, it can be determined that K709 plays a major role in the degradation of HIF-1α through SIRT1 inactivation.

### VHL binds to and degrades acetylated HIF-1α in hypoxia

**Fig. 6:**
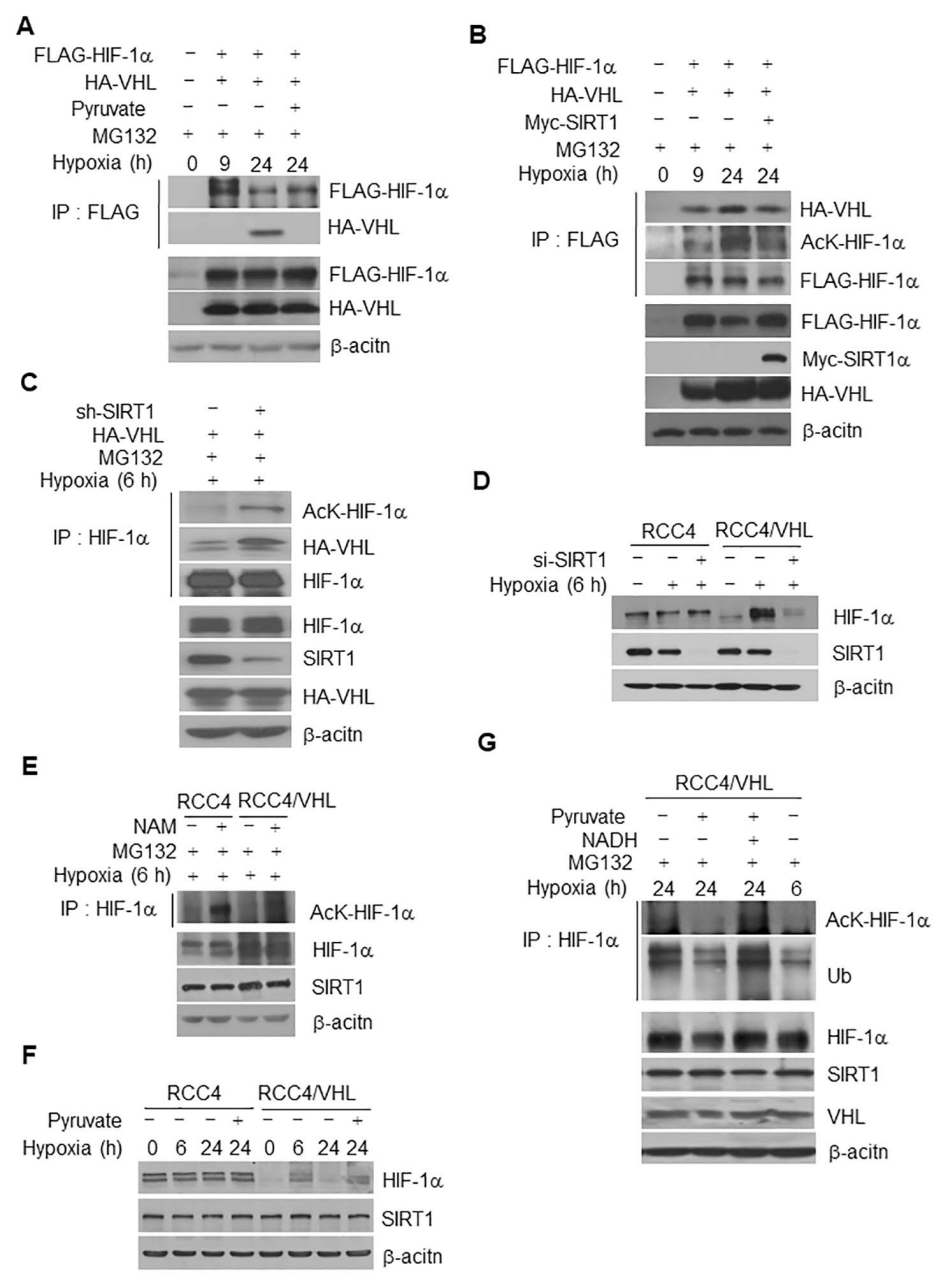
The extent of HIF-1α acetylation and HIF-1α interaction with VHL is increased during chronic hypoxia via SIRT1 inactivation. (**A ~ C**) HeLa cells co-transfected with *Flag*-tagged *HIF-1α* and either *HA*-tagged *VHL* (*HA*-*VHL*) were exposed to hypoxic conditions for 9 and 24 h (**A** and **B**) or 6 h (**C**). The effect of pyruvate (**A**), *Myc*-tagged *SIRT1* (**B**), and either *SIRT1*- or control-shRNA (-) transfection (**C**) on HIF-1α interaction to VHL (**A** ~ **C**), acetylation, (**B** and **C**) was monitored in immunoprecipitated HIF-1α preparations, followed by probing with anti-acetyl-K and/or anti-HA antibodies, respectively. (**D** ~ **E**) The contribution of an association of VHL with HIF-1α in terms of HIF-1α stability was determined in the presence of *SIRT1*- (+) or control-siRNA (-), or pyruvate in RCC4 and RCC4/*VHL* cells. (**E**) The extent of acetylation of immunoprecipitated HIF-1α was assessed in RCC4 and RCC4/*VHL* cells treated with NAM. (**F-G**) Acetylation and ubiquitination of immunoprecipitated HIF-1α were assessed in RCC4/*VHL* cells treated with pyruvate alone (**F**), or with concomitant addition of NADH 6 h before harvest (**G**). The concentration of MG132, pyruvate and NADH was 10 mM, 1 mM, and 1 mM, respectively

Degradation of HIF-1α results from interaction with VHL E3 ligase under both normoxic and hypoxic conditions [50–53]. We initially analyzed the interaction of HIF-1α and VHL in 293T cells in order to determine whether degradation of HIF-1α induced by chronic elevation of the NADH concentration and subsequent inactivation of SIRT1 is associated with VHL. According to the results of immunoprecipitation analysis, HA-tagged VHL was specifically detected in Flag-tagged HIF-1α immunoprecipitates 24 h after hypoxic exposure, but not 9 h (Fig. 6A), indicating that interaction of HIF-1α with VHL occurs under conditions of chronic hypoxia. This was accompanied by increased ubiquitination (Fig. S7A). The addition of pyruvate resulted in dissociation of the HIF-1α-VHL interaction and -ubiquitination under conditions of chronic hypoxia (Fig. 6A and Fig. S7A) caused by decreased acetylation of HIF-1α (Fig. 4A and B) and an increase in the concentration of NADH (Figs. 1A and 3B). Overexpression of SIRT1 consistently reduced the extent of the chronically increased acetylation, interaction with VHL (Fig. 6B), and ubiquitination of HIF-1α (Fig. S7B). Based on these data, interaction of HIF-1α-VHL under conditions of chronic hypoxia appears to be due to increased acetylation of HIF-1α. To confirm this as fact, we further examined the effect of SIRT1 on the interaction of HIF-1α-VHL during an early phase of hypoxia. Depletion of SIRT1 caused an increase in the level of HA-tagged VHL in HIF-1α immunoprecipitate, accompanied by increased acetylation (Fig. 6C) and ubiquitination (Fig. S7C) of HIF-1α during hypoxic exposure.

Obvious degradation of HIF-1α caused by depletion of SIRT1 was not observed in RCC4 cells (*VHL*-null) during the early phase of hypoxia, in contrast to that observed in *VHL*-rescued RCC4/*VHL* cells (Fig. 6D). However, increased acetylation of HIF-1α after inhibition of SIRT1 was observed in RCC4 (*VHL*-null) and *VHL*-rescued cells (Fig. 6E), indicating that VHL is required for degradation of hypoxic HIF-1α via an increase in acetylation. Similar to the result for early hypoxia, chronic degradation of HIF-1α during 24 h hypoxia also occurred in *VHL*-rescued RCC4 cells but not RCC4 cells (Fig. 6F). *VHL*-rescued RCC4 cells responded to pyruvate by inhibiting degradation (Fig. 6F), acetylation, and ubiquitination of HIF-1α (Fig. 6G) that were elevated during chronic hypoxia. Addition of NADH repeatedly resulted in elimination of the pyruvate effect via re-elevation of acetylation and ubiquitination (Fig. 6G). Based on these results, we can conclude that SIRT1-mediated protection of HIF-1α acetylation and degradation is attenuated by a signaling process resulting from upregulation of NADH levels, in turn causing VHL-dependent degradation of HIF-1α.

### Chronic degradation of HIF-1α by acetylation is independent of proline hydroxylation

**Fig. 7:**
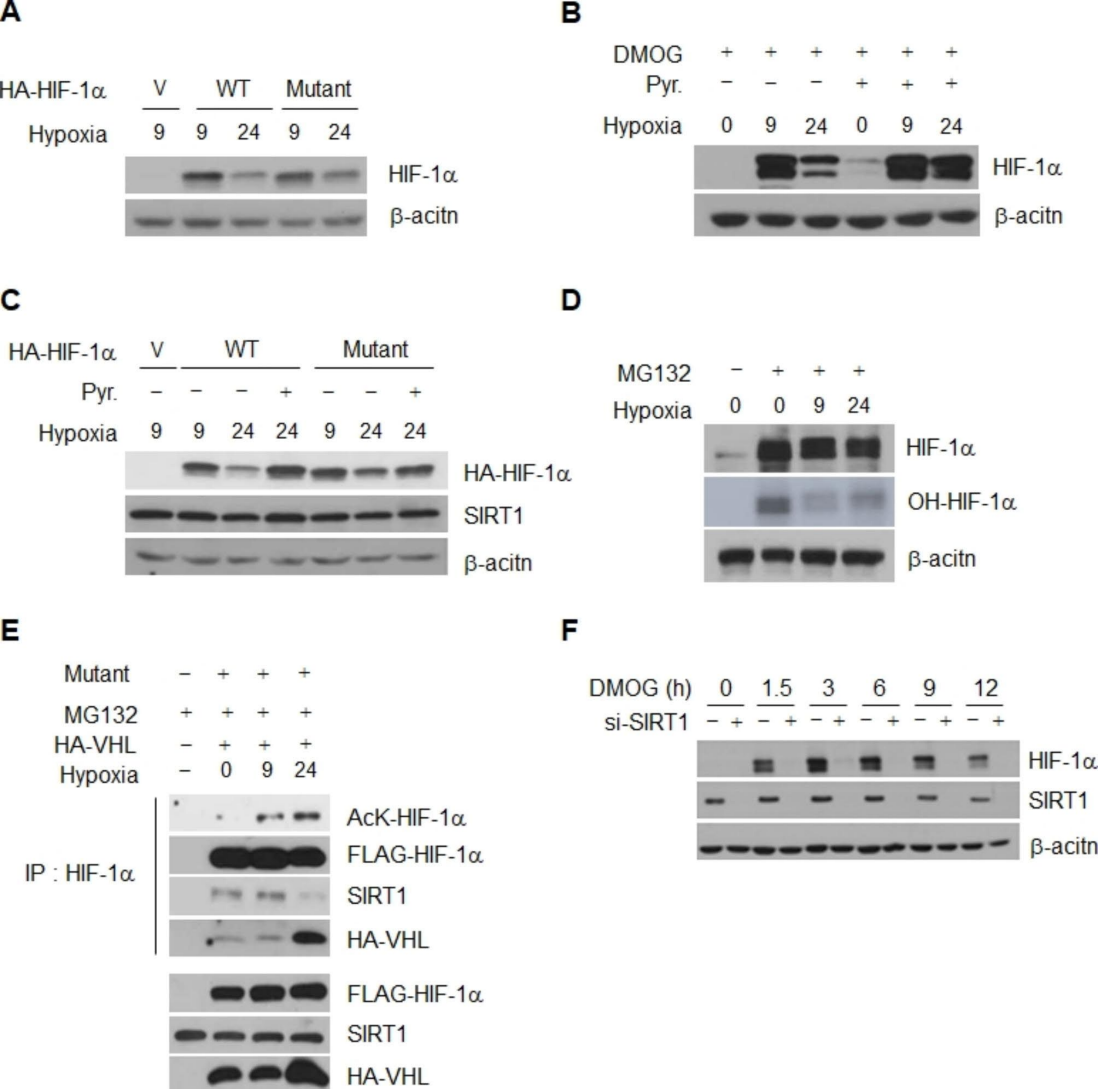
Chronic degradation of HIF-1α is independent of proline hydroxylation. HeLa cells were transiently transfected with plasmids encoding *HA*-tagged wt-*HIF-1α* (WT), proline mutant *P402A/P564A* (Mutant) and empty vector (V), or treated with DMOG (0.5mM). (**A** ~ **C**) The levels of the exogenous wt- and mutant-HIF-1α proteins **(A)**, and the DMOG-exposed endogenous HIF-1α were determined 9 and 24 h after commencement of hypoxia (**B**). The effect of pyruvate on the exogenous wt- and mutant-HIF-1α proteins HIF-1α levels was examined (**C**). The HIF-1α hydroxylation (OH-HIF-1α) (**D**), and the mutant acetylation and interaction to HA-tagged VHL in HIF-1α immunoprecipitates (**E**) were measured in the presence of MG132 under acute and chronic hypoxia condition. (**F**) At the indicated times after DMOG addition, HIF-1α levels were compared between HeLa cells with *SIRT1*- (+) or control-siRNA transfection (-) under normoxic condition. The concentration of pyruvate and MG132 was 1 mM and 10 mM, rescpectively

Detachment of VHL from HIF-1α occurs via dehydroxylation at two proline residues, Pro402 and Pro564, which in turn leads to stabilization of the protein [50–53]. To determine whether inactivation of SIRT1 and consequent degradation of HIF-1α during chronic hypoxia occur in association with proline hydroxylation, we initially examined the stability of a proline mutant HIF-1α (P402A/P564A) where proline 402 and 564 are replaced with arginine; this mutant is defective in hydroxylation and resistant to degradation under conditions of normoxia (Fig. S8). However, a decrease in the amount of the transfected mutant HIF-1α (P402A/P564A) was observed 24 h after commencement of hypoxia and the same result was observed for wild-type HIF-1α (Fig. 7A and Fig. S8). In addition, treatment with DMOG, a proline hydroxylase inhibitor, resulted in chronic degradation of HIF-1α (Fig. 7B), suggesting that even degradation of dehydroxylated HIF-1α can occur under conditions of chronic hypoxia. However, the amount of the chronically degraded P402A/P564A mutant as well as -wt-HIF-1α and -DMOG-exposed HIF-1α was recovered by addition of pyruvate (Fig. 7B and C). In addition, a similarly low level of HIF-1α hydroxylation was observed at 9 and 24 h after commencement of hypoxia (Fig. 7D), indicating that chronic degradation of HIF-1α is independent of proline hydroxylation under our culture conditions. Consistent with the results observed for chronic degradation (Fig. 7A and S8), the mutant P402A/P564A showed an increase in acetylation and interaction with VHL 24 h after the commencement of hypoxia (Fig. 7E). The results imply that acetylation and degradation of the proline mutant also occurs under conditions of chronic hypoxia via recruitment of VHL as with wild-type HIF-1α. In addition, the stabilization of HIF-1α induced by treatment with DMOG was also inhibited by depletion of SIRT1 under conditions of normoxia (Fig. 7F), indicating that, even under the condition of inhibition of hydroxylation, SIRT1 is still required for stabilization of HIF-1α. Our current data suggest that inhibition of SIRT1 and consequent acetylation-induced degradation of HIF-1α during the chronic phase is independent of proline hydroxylation. Both proline dehydroxylation and SIRT1-mediated deacetylation of HIF-1α are required for stabilization during the acute phase.

### Chronic degradation of HIF-1α weakens invasion of cancer cells via SIRT1 inactivation

**Fig. 8:**
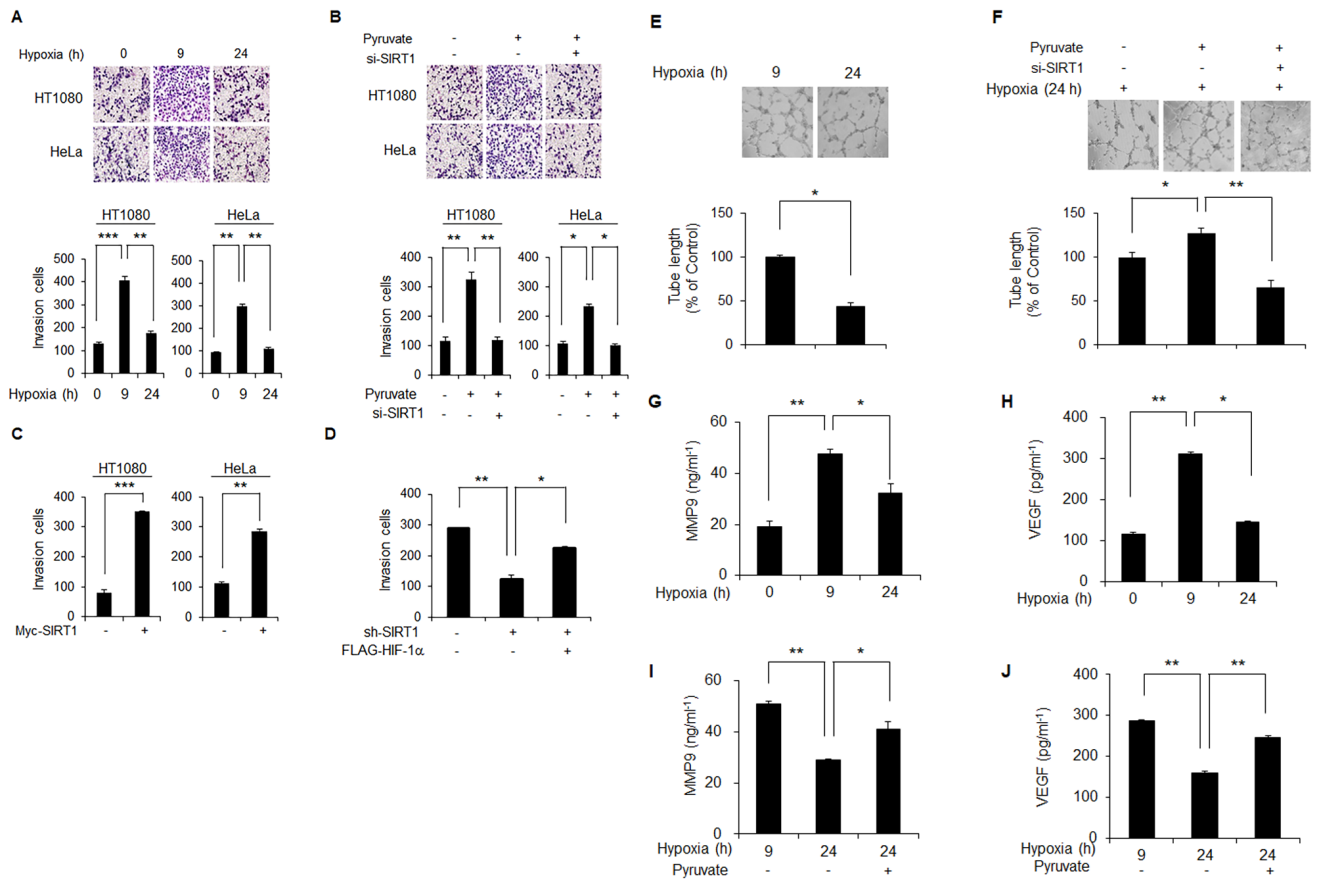
Chronic degradation of HIF-1α attenuates invasive and angiogenesis properties of cancer cells under hypoxic conditions. (**A-D**) Invasion activity of HeLa and HT1080 cells growing under acute and/or chronic hypoxia over 9 and/or 24 h, respectively, was determined using a matrigel invasion chamber **(A)**. Invasion activity under chronic hypoxia was determined in the absence and presence of either pyruvate alone or together with *SIRT1*-siRNA (**B**) or in the presence of *Myc*-tagged *SIRT1***(C)** or sh-SIRT1 alone or in combination with HIF-1 (**D**). SIRT1 depletion was achieved with three different *SIRT1*-siRNAs (lower panel, **B**). Invasion activity of HeLa cells with sh-control (-) or sh-*SIRT1* (+) plasmid was determined after transfection of either *Flag*-tagged *HIF-1α* (+) or empty vector (-). (**E** and **F**) Angiogenic activity of HeLa cells growing under acute and chronic hypoxia over 9 and 24 h, respectively, was determined by measuring tube length of HUVEC cells in the absence and presence of either pyruvate alone or together with *SIRT1*-siRNA (+) or control-siRNA (-) (**F**). (**G**-**J**) The amounts of MMP9 and VEGF proteins secreted into culture medium were determined in HeLa cells growing under hypoxia over 9- and 24-h in the absence and presence of pyruvate. The concentration of pyruvate was 0.05 mM. Statistical significance (*p*-value) was determined using the ANOVA-t-test. * : *p* < 0.05, ** : *p* < 0.01, *** : *p* < 0.001

We initially compared the rate of invasion between cells with 9- and 24-h hypoxic exposures in order to determine the biological consequences of SIRT1-mediated chronic degradation of HIF-1α. A significantly lower rate of invasion was observed in 24 h-hypoxia-exposed cells with chronic degradation of HIF1-alpha compared to those with 9 h-hypoxic exposure and thus accumulation of HIF-1α (Fig. 8A). The capacity for invasion that was diminished during the chronic phase of hypoxia was recovered by either addition of pyruvate (Fig. 8B) or *Myc*-tagged transfection of *SIRT1* (Fig. 8C). However, pyruvate-mediated recovery of the chronically decreased invasion was not observed in SIRT1-depleted cells (Fig. 8B). In fact, a signifcanlty lower rate of invasion was observed in SIRT1 knockdown cells compared to those with control-siRNA, which was confirmed by three distinct *SIRT1*-siRNAs (Fig. S9). To further confirm the effect of HIF-1α on invasion during chronic hypoxia, transfection of *Flag*-tagged *HIF-1α* was performed for rescue of HIF-1α in SIRT1-depleted cells. Restoration of HIF-1α in SIRT1-depleted cells resulted in recovery of the diminished invasion activity (Fig. 8D). Similar to the results for invasion, significant recovery of the angiogenic activity that was decreased during chronic hypoxia (Fig. 8E) was achieved by addition of pyruvate (Fig. 8F). In addition, the pyruvate effect on angigenic activity was not observed under the condition of SIRT1 depletion (Fig. 8F). Next, measurement of MMP2 and VEGF proteins was performed in order to determine whether the invasion and angiogenic activities that were diminished during chronic hypoxia was associated with transcriptional activity of HIF-1α. As expected, increased expression of MMP2 and VEGF observed during the acute phase of hypoxia was decreased during the chronic phase (Fig. 8G and H). The chronically decreased expression of VEGF and MMP2 was recovered by the addition of pyruvate (Fig. 8I and J). These data suggest that invasion by cancer cells is promoted by activation of SIRT1 during the early phase of hypoxia via accumulation of HIF-1α; this invasion activity is reduced during chronic hypoxia via degradation of HIF-1α attributable to elevation of NADH and subsequent inactivation of SIRT1.

## Discussion

In the current study, we propose a model explaining regulation of HIF-1α by SIRT1 under conditions of chronic hypoxia as a metabolic sensor and switch for detection of harsh environmental condtions without oxygen and nutrients. Based on our findings, elevation of the NADH concentration promotes degradation of HIF-1α via inactivation of SIRT1 and increased acetylation of HIF-1α under conditions of chronic hypoxia. Specifically, we suggest that the acetylated HIF-1α lysine 709 is recognized by VHL, leading to degradation via ubiquitin/proteasome machinary under conditions of chronic hypoxia, as with proline-hydroxylated HIF-1α under conditions of normoxia. In addition, attenuation of the agigogenic and invasion activities of cancer cells that are strengthened under acute hypoxic conditions occurs along with degradation of HIF-1α during chronic hypoxia.

Although cancer cells show progressive aggressiion under conditions of hypoxia, some differences in their characteristics between hypoxic conditions during acute and chronic phases have been reported. For example, radiation-resistance, invasion [11–14] and metastasis activities [54, 55] are milder during chronic hypoxia compared with acute-hypoxia. Many reports have suggested that switching of roles between HIF-1α and HIF-2α in transition from conditions of acute to chronic hypoxia leads to development of these phenotypic differences [56, 57]. Indeed, the level of HIF-1α protein peaks at around 4–8 h and shows a continuous decline thereafter, while HIF-2α shows continuous stability until reaching the stage of chronic hypoxia [20, 57–60].

After clarifying the mechanistic basis of HIF-1α degradation during chronic hypoxia, we believed that our current finding establishes the mechanistic basis for these preceeding reports. Indeed, such behaviors of cancer cells are controlled by HIF-1α. However, stronger aggressiveness can be observed under conditions of chronic hypoxia compared with those of acute hypoxia in vitro [8]. The descrepancy in biological consequences between the reports would be a result of differences in experimental conditions including the method of hypoxia induction, oxygen and nutrient concentrations, cancer cell type etc. [22, 60].

Under hypoxic conditions tumor cells offen have restricted access to nutrients and oxygen, which affects many cellular metabolic systems including glucose metabolism [61]. Under chronic hypoxic conditions, excessive accumulation of lactic acid occurs via exhaustion of pyruvate, which is catalyzed by lactate dehydrogenase [62, 63]. Excess lactic acid slows or prevents further reduction and oxidation of pyruvate and NADH, which appears to lead to elevation of cellular NADH content [64]. Based on these facts, the nutrient ingredients and content of culture media have a significant effect on experimental results in the in vitro system. Under our minimal culture conditions using Minimal Essential Medium (MEM) without pyruvate, we demonstrated that SIRT1 functions as a major metabolic switch in detection of chronically elevated NADH and transmits this information to induce weakening of HIF-1α stability via an increase in acetylation. To date, thorough research on regulation of HIF-1α activity by SIRT family members under chronic hypoxic conditions has not been conducted, although several connection studies on stabilization of HIF-1α, including SIRT1 [24, 25], SIRT2 [26], SIRT3 [27, 28], and SIRT6 [29] have been reported. However, reports on regulation of HIF-1α by SIRT1 have been controversial, initially reporting either no impact [30] or a negative impact [25] on HIF-1α, however, studies reporting positive effects, including ours, were reported later [24, 32]. A previous study conducted by Lim et al. reporting on negative control of the transcriptional activity of HIF-1α by SIRT1 did not assess HIF-1α stability [25]. The authors used Dulbecco’s Modified Eagle’s Medium (DMEM) containing a high level of pyruvate for cultivation of HT1080, HEK293T, and HCT116. As demonstrated in our current results as well as those of other studies, hypoxic elevation of NADH was prevented by addition of pyruvate [65, 66]. In fact, degradation of HIF-1α was not observed within 48 h after the commencement of hypoxia in HeLa and HT1080 cells cultured in DMEM, unlike the results obtained using MEM without pyruvate (Fig. S1A). These results indicate that the increase of NADH is prohibited by pyruvate in DMEM, consequently sustaining the activity of SIRT1 under conditions of chronic hypoxia.

Although decomposition of HIF-1α by VHL in nomoxia is known to occur, few studies on the stability of HIF-1α protein under conditions of hypoxia have been reported. However, findings from recent studies have demonstrated that the expression level of the HIF-1α protein is gradually decreased, unlike HIF-2α, which is maintained in a hypoxic state [67]. In addition, the fact that the decline in expression of HIF-1α is mainly due to decreased protein stability, not regulation of transcription, has been demonstrated [12, 60, 68]. In this study, we first demonstrated a novel mechanism for control of HIF-1α stability via SIRT1/VHL signaling under conditions of hypoxia as well as those of normoxia. Our finding provided further resolution to a longstanding question: why does destruction of HIF-1α in *VHL*-deficient renal cancer cells not occur during chronic hypoxia [69].

After further consideration of the interaction between SIRT1 and HIF-1α it was determined that comparison of the phenotypes of HIF-1α- and *SIRT1*-knockout mice was appropriate. HIF-1α knockout is lethal in mice on embryonic day 9.5 due to failure of vascularization [70]. Thus we expected that *SIRT1* knockout would also be embryonically lethal, because of impaired stabilization of HIF-1α and subsequent development of a defect in vascularization during embryogenesis. However, progression of *SIRT1* knockout mouse embryos to birth has been reported [71]. These paradoxical findings suggest the possible existence of a pathway involving SIRT1-independent stabilization of HIF-1α, or that HIF-1α-independent vascularization may occur under conditions of SIRT1 depletion. Indeed, partial or extensive stabilization of HIF-1α was observed under conditions of SIRT1 inactivation, respectively (Supplementary Figure S11D). HIF-1α-independent vascularization has also been observed under several different sets of physiological conditions [72]. Therefore, further evaluation of these findings will be necessary in order to understand the mode of HIF-1α stabilization involving SIRT1. In this study, we elucidated a distinct mechanism governing HIF-1α degradation in chronic hypoxic cancers, proposing its variability based on cancer metabolism and VHL activity. Our findings indicate that such selectivity in HIF-1α expression within chronic hypoxia might contribute to distinctive cancer traits. However, research on cancer characteristics linked to HIF-1α in chronic hypoxia remains limited, underscoring the need for further investigation.

## Conclusions

**Fig. 9:**
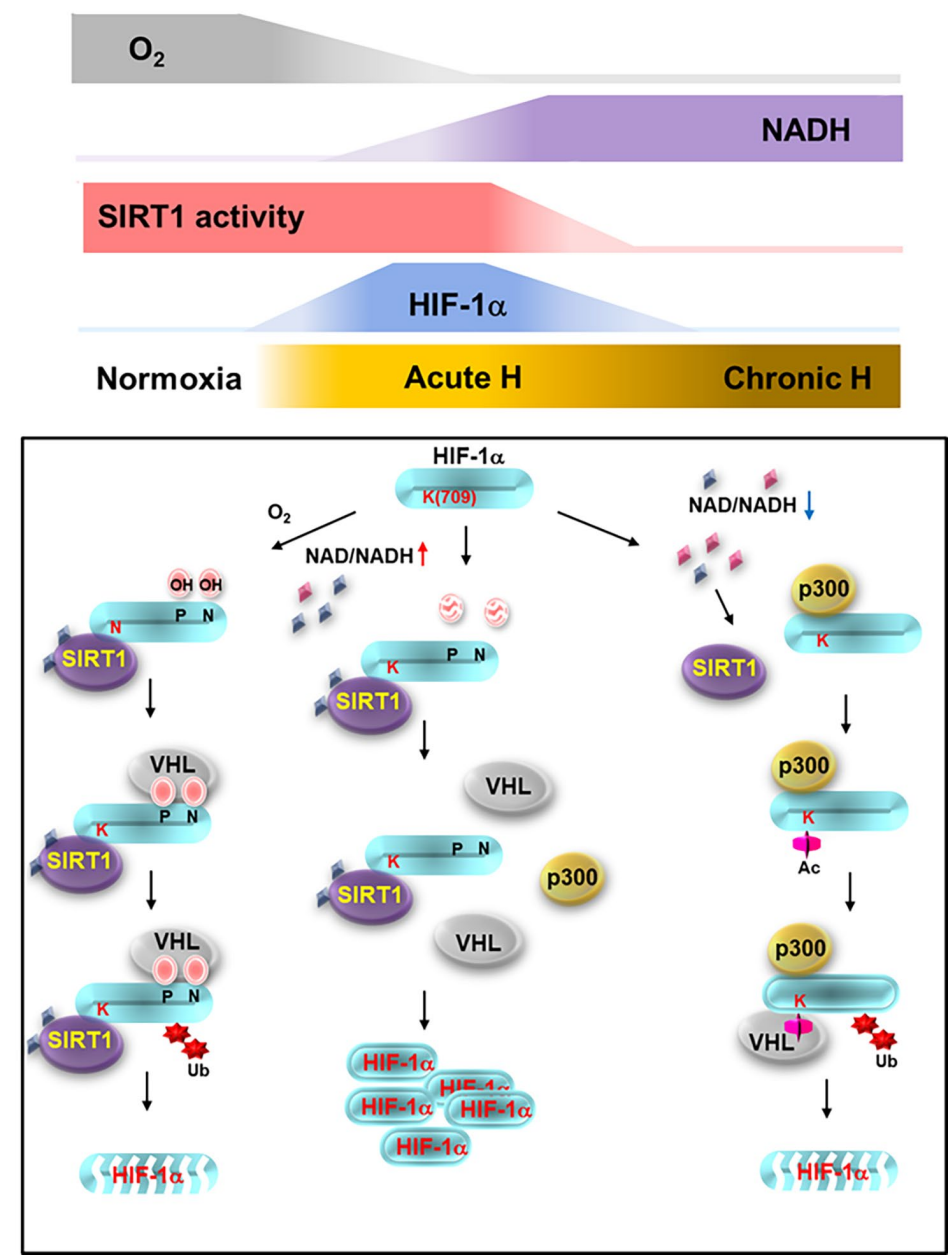
A schematic showing how sensing by the redox couple NAD^+^/NADH acetylation mediates HIF-1α degradation and stabilization, employing SIRT1 during acute and chronic hypoxia. During normoxia, oxygen-sensing HIF-1α prolines, Pro402 and Pro564, are hydroxylated by HIF hydroxylases, promoting HIF-1α degradation in an oxygen-sensitive manner. HIF-1α degradation occurred during chronic hypoxia, however, is controlled in an NADH-sensitive manner via acetylation of the protein. NADH, the level of which is upregulated during chronic hypoxia, functions as a destabilizing messenger for chronic HIF-1α decay. Surplus NADH inactivates SIRT1 (iSIRT1), and HIF-1α interaction with VHL that is attenuated after commencement of hypoxia is thus renewed. This leads to elevated ubiquitination and degradation of HIF-1α. However, during the acute phase of hypoxia, NAD^+^ functions as a stabilizing messenger; the NAD^+^-sensing protein SIRT1 (aSIRT1) protects HIF-1α from redox-sensitive acetylation, thereby facilitating dissociation of HIF-1α from VHL and initiating HIF-1α stabilization

The findings of our study suggest a new model for inactivation of SIRT1 resulting from elevated NADH in chronic hypoxia, which enables acetylation of HIF-1α by p300. In addition, we discovered a new role of VHL in degradation of HIF-1α via recognition of the acetylated protein in chronic hypoxia. Defining the regulation of HIF-1α protein stability in chronic hypoxia can be helpful in development of a strategy for determining our focus in the ongoing changes of status that occur during hypoxia. In addition, our findings will serve as an important biological milestone that will change the established belief that involvement of conventional VHL in degradation of HIF-1α occurs only in the normoxia state (Fig. 9).

## Materials and methods

### Cell culture, reagents, and treatment

HeLa and HT1080 cells were cultured in Minimal Essential Medium (MEM; Cat. no. LM007-7, Welgene, Daegu, Korea) supplemented with 10% (v/v) fetal bovine serum (FBS; Cat. no. 43640, JRS, CA) and 1% (w/v) penicillin/streptomycin. RCC4 and RCC4/VHL cells (kindly provided by Dr. Bernard Brüne) [73] were maintained in Minimal Essential Medium with 10% FBS. HEK293T cells were maintained in Dulbecco’s Modified Eagle’s Medium (DMEM; Cat. no. LM001-05, Welgene) with 10% (v/v) FBS and antibiotics. A stable HeLa cell line with depleted SIRT1 (HeLa-shSIRT1) was used, which had already been established in a previous study [22]. The cells were cultured in a 5% CO2 chamber at 37 °C. To expose hypoxic conditions, cells were incubated in a hypoxic chamber (Forma Anaerobic System; Thermo Scientific, MA) with 5% CO_2_/0.5% or 1.0% O_2_ and 94.5% or 94.0% N_2_ (all v/v), respectively.

The following reagents were used in this study: nicotinamide (NAM; Cat. no. z40206, Sigma, St. Louis, MO), MG132 (Cat. no. C2211, Sigma), nicotinamide adenine dinucleotide (NAD^+^; Cat. no. N7004, Sigma), reduced nicotinamide adenine dinucleotide (NADH; Cat. no. N8129, Sigma), sodium pyruvate (Cat. no.P5280, Sigma), CoCl_2_ (Cat. no.C8661, Sigma), EX-527 (Cat. no. 2780, Tocris, Bristol, UK), lactate (Cat. no. L1750, Sigma), nicotinic acid (Cat. no.N0761, Sigma), and dimethyloxaloylglycine (DMOG) (Cat. no.D3695, Sigma).

### Plasmid, siRNA, transfection

Plasmids encoding Flag- and HA-tagged wt-HIF-1α including proline mutant (P402A/P564A) were kindly provided by Dr. Lorenz Poellinger [74] and Dr. Jong-Wan Park [75], respectrively. HA-tagged VHL was a gift from Youn H. D [76]. Wt-SIRT1 and dominant negative SIRT1/H363Y were acquired from Weinberg R. A [44]. HA-Ubiquitin was a gift from Edward Yeh (Addgene plasmid # 18712). Construction of the sh-SIRT1 plasmid was performed using primer sets (Table S1) and the annealed primer was cloned into the pENTP^TM^/H1/TO vector. For overexpression, the 3ug of target vector or its corresponding backbone vector was transfected into cells cultured on 60 mm dish using Turbofect transfection reagent according to the manufacturer’s protocol (Fermentase, Ontario, Canada).

For gene silencing, transfection of cells with Lipofectamine RNAi MAX (Cat. no. 13778-150, Invitrogen) was performed according to the manufacturer’s instructions. A final concentration of 20nM of either negative control or target siRNA was utilized. The sequences of oligonucleotides used in siRNA experiments were as follows: si-control (Cat. no. 12935300; Invitrogen); si-SIRT1 (Table S1); si-NAMPT (Cat. no. 1,112,709, 1,112,710; Bioneer, South Korea); and si-AMPK (Cat. no. 1,121,714, 1,121,715; Bioneer).

### Immunoblotting

TNN buffer (40mM Tric-HCl (pH8.0), 120mM NaCl, 0.5% NP-40) supplemented with 1mM phenylmethylsulfonyl fluoride, 100mM sodium fluoride and protease inhibitors was used for lysis of cells (Cat. no. 04693159001, Roche, Penzberg, Germany). SDS-PAGE was performed for separation of cell extracts, followed by transfer to nitrocellulose membranes. Immunoblotting of membranes was performed using anti-SIRT1, anti-HA, anti-c-Myc, anti-β-Actin (Cat. no. sc-55,404, sc-805, sc-40, sc-47,778, Santa Cruz Biotechnology, CA), anti-HIF-1α, anti-VHL (Cat. no. 610,959, 556,347, BD Biosciences, NJ), or anti-Flag antibody (Cat. no. F3165, Sigma), followed by incubation with the appropriate horseradish peroxidase (HRP)-conjugated secondary antibodies. These included anti-mouse IgG (H^+^L)-HRP and anti-rabbit IgG (H^+^L)-HRP (Cat. no. K0211589, K0211708, Koma, Seoul, Korea). Chemiluminescent detection was performed using the ECL system (Cat. no. RPN 2106, Amersham, HP7 9NA,UK).

### Protein-protein interaction assays

Lysis of cells was performed using TNN buffer followed by preclearing using normal mouse IgG (Cat. no. sc-2025, Santa Cruz Biotechnology). Lysates were immunoprecipitated with either normal rabbit IgG (Cat. no. sc-2027, Santa Cruz Biotechnology), normal mouse IgG, anti-SIRT1, or anti-HIF-1α antibody (Cat. no. NB100-105, Novus Biologicals) for detection of the interaction between HIF-1α and SIRT1. An HA-tagged VHL expression plasmid [76] was used in cotransfections with Flag-tagged HIF-1α for evaluation of VHL binding to HIF-1α.

### Acetylation and ubiquitination assays

Lysates for detection of acetylation of both endogenous and exogenous HIF-1α were prepared at the indicated time points from cells exposed to hypoxia and from cells transfected with Flag-tagged HIF-1α constructs. These lysates were subjected to immunoprecipitation with anti-HIF-1α (Cat. no. NB100-105, Novus Biologicals) or anti-FLAG antibody for measurement of the acetylation levels of endogenous or exogenous HIF-1α, respectively. Immunoblotting using an anti-acetyl-lysine (AcK) antibody (Cat. no. 9441, Cell Signaling, MA) that specifically recognizes acetylated lysines was performed for detection of acetylated HIF-1α. The ratio of acetylated HIF-1α/HIF-1α band intensities was calculated for quantitation of the extent of HIF-1α acetylation. For ubiquitination assays, immunoprecipitation of cell lysates from cotransfectants expressing HA-tagged ubiquitinase and FLAG-tagged HIF-1α was performed using anti-Flag antibody, followed by resolution using SDS-PAGE. For detection of ubiquitin, the blotted membranes were sandwiched between several sheets of Whatman 3 M paper and submerged in deionized-water. Membrane-bound ubiquitin was then heat-activated by autoclaving for 30 min. Immunoblotting using anti-HA and anti-Flag antibodies was performed for determination of the levels of ubiquitination and HIF-1α, respectively.

### Measurement of intracellular NAD^+^ and NADH levels

Measurement of intracellular NAD^+^ and NADH levels was performed using NAD^+^/NADH enzyme cycling as as previously described [46, 77–79]. Enzyme cycling assays were performed according to the manufacturer’s protocol (Cat. no.K337-100, Biovision, CA). In brief, extraction of cells (2.0 × 10^5^) was performed using NADH/NAD extraction buffer during homogenization. Each extract was equally divided into two tubes; one tube was used to determine total NAD, and the other total concentrations of NADH. NAD^+^ was decomposed by heating at 60 °C for 30 min for detection of NADH. Measurement of optical density was performed at 450 nm for determination of NADH levels. Means and standard deviations of NAD^+^ and NADH concentrations were obtained from triplicate samples. Evaluation of the concentrations of NAD^+^ and NADH was performed by calculating their amounts per sample of 50 µg protein applied, within the linear range of nicotinamide nucleotide and sample standard plots.

### Semi-quantitative RT-PCR analysis

Extraction of total cellular RNA was performed using an RNeasy mini kit (Cat. no. 74106, Qiagen, CA). Reverse transcription of total RNA (1 µg) was performed using SuperScript II reverse transcriptase (Cat. no. 18064, Invitrogen) and an oligo (dT) primer (Cat. no. 18418012, Invitrogen). One-tenth of the cDNA from the first-strand reaction was amplified using a Maxime PCR premix kit (Cat. no. 25167, iNtRON Biotechnology). The PCR primers used for detection of mRNA levels are shown in Supplementary Table S2.

### Real-time RT-PCR analysis

Total RNA (1 µg) was used in reverse transcription reactions using an oligo-dT primer (Cat. no. 58,862, Invitrogen). Real-time RT-PCR was performed in a volume of 20 µL containing one-tenth of the cDNA sample, 2x SYBR premix Taq (Cat. no. 1708880, Bio-Rad, CA), and appropriate primers (shown in Table S2**)**. The levels of *HIF-1α* and *SIRT1* transcription were normalized to the level of *β-Actin*. Amplification was performed in two steps: pre-incubation at 95 °C for 3 min followed by 45 cycles at 95 °C for 15 s, 60 °C for 20 s, and 72 °C for 30 s. Each RT-PCR reaction was repeated at least three times in order to demonstrate reproducibility, and analysis of data was performed using the CFX96 Real-Time System (Bio-Rad). Each normalized value was obtained by subtracting the threshold cycle (Ct) of *β-Actin* from the Ct values of target genes, yielding ∆Ct numbers, and the formula ∆∆Ct was used as an indication of the relative transcriptional level.

### Invasion

Analysis of cellular invasion activity was performed using a transwell chamber (Cat. no. 3422, Corning, NY) in which inserts were coated with dilutions of BD Matrigel Matrix Growth Factor Reduced (BD Biosciences, Bedford, MA) HeLa and HT1080 cells were seeded into the upper wells at a density of 1 × 10^4^ cells per well in serum-free MEM, followed bv incubation for 24 h under either normoxic or hypoxic conditions. Cells invading the lower surface membrane were fixed and stained with Hemacolor solution (Cat. No. 1.11661 Merck, Darmstadt, Germany). The numbers of mobile cells were counted under a light microscope to determine the extent of invasion.

### Electronic supplementary material

Below is the link to the electronic supplementary material.

Supplementary Material 1

Supplementary Material 2

Supplementary Material 3

## Data Availability

The datasets supporting the conclusions of this article are included within the article and its additional files.
