# Supplementary material for: NADH elevation during chronic hypoxia leads to VHL-mediated HIF-1α degradation via SIRT1 inhibition
Source: Cell Biosci. 2023 Sep 30;13:182. doi: 10.1186/s13578-023-01130-3 (PMC10543270; doi:10.1186/s13578-023-01130-3)
Supplement: Supplementary file 1 — Supplementary Material 1 [file 13578_2023_1130_MOESM1_ESM.pptx]

## Slide 1
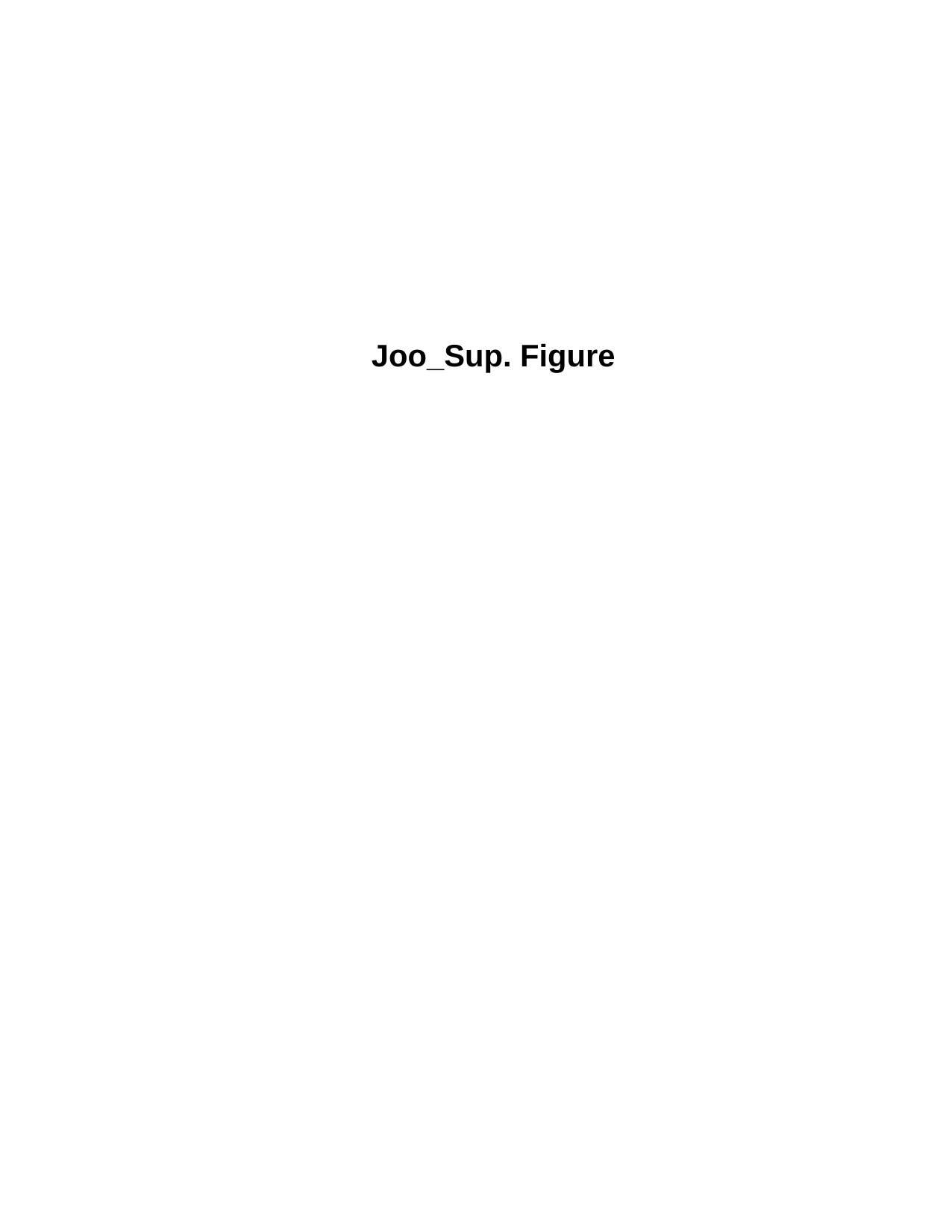

Joo_Sup. Figure

## Slide 2
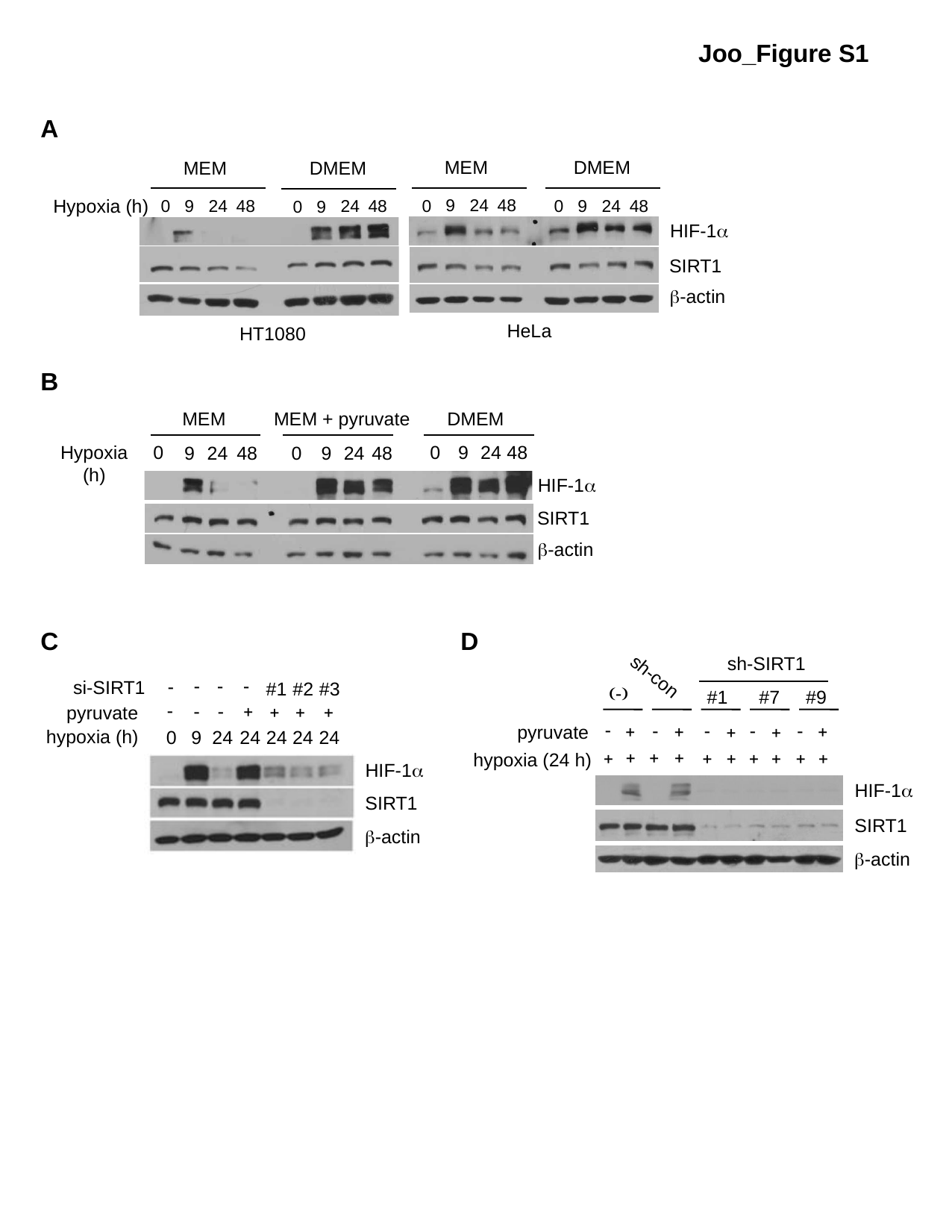

Joo_Figure S1
A
DMEM
MEM
DMEM
MEM
Hypoxia (h)
24
48
9
0
24
48
24
48
9
0
9
0
24
48
9
0
HIF-1a
SIRT1
b-actin
HeLa
HT1080
B
MEM + pyruvate
DMEM
MEM
0
9
24
48
0
0
9
24
48
9
24
48
HIF-1a
SIRT1
b-actin
Hypoxia (h)
C
D
sh-SIRT1
sh-con
si-SIRT1
-
-
-
-
#2
#3
#1
-
+
-
-
+
+
+
pyruvate
hypoxia (h)
0
24
24
24
24
24
9
HIF-1a
SIRT1
b-actin
(-)
#1 #7 #9
-
pyruvate
+
+
-
-
-
+
-
+
+
+
+
+
+
+
+
+
+
+
+
hypoxia (24 h)
HIF-1a
SIRT1
b-actin

## Slide 3
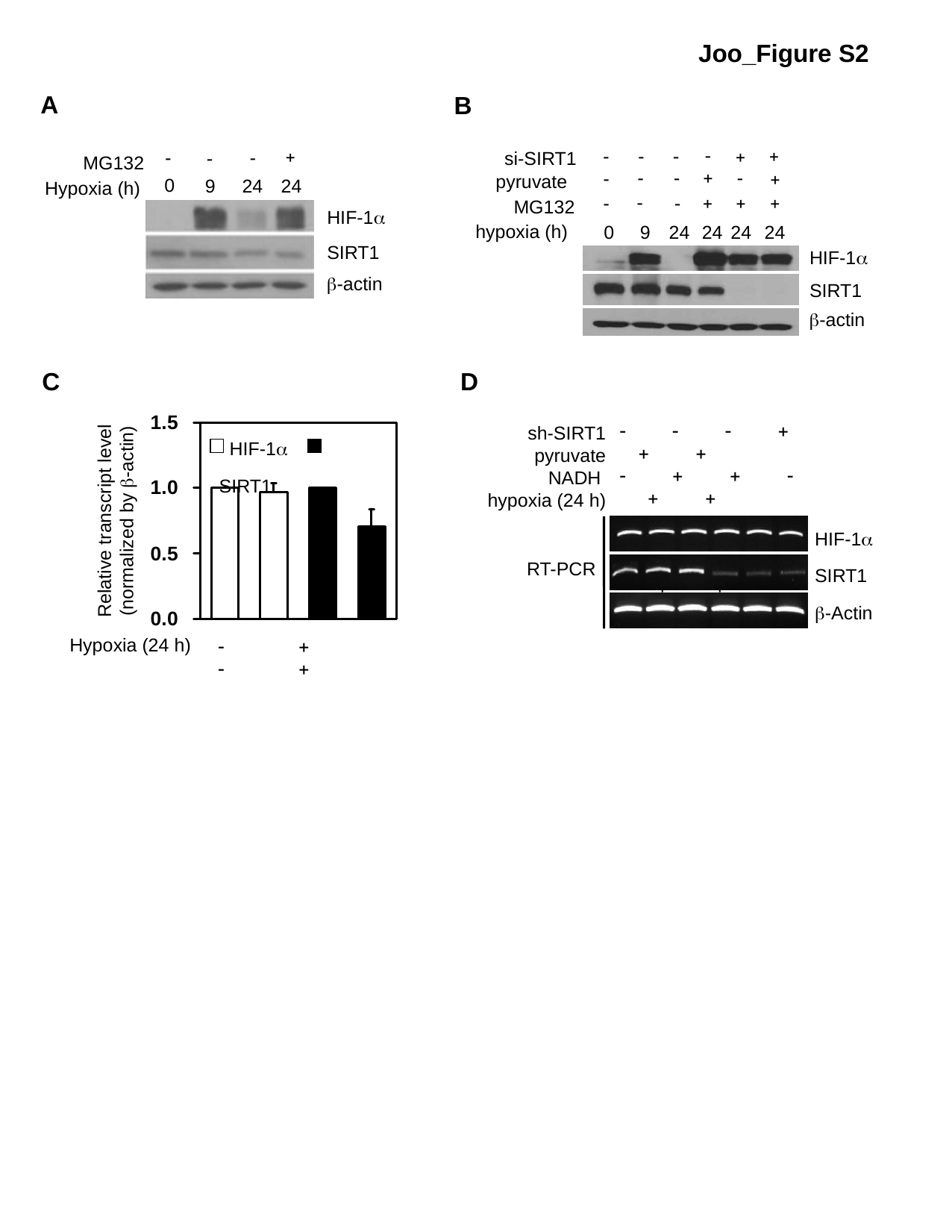

Joo_Figure S2
A
B
-
+
-
+
+
-
-
-
MG132
0
24
24
9
Hypoxia (h)
HIF-1a
SIRT1
b-actin
-
-
si-SIRT1
+
-
-
-
+
-
pyruvate
-
+
+
+
-
-
MG132
hypoxia (h)
0
9
24
24
24
24
HIF-1a
SIRT1
b-actin
C
D
- - - + + +
- + + - + +
- - + - - +
+ + + + + +
sh-SIRT1
pyruvate
NADH
hypoxia (24 h)
 HIF-1α SIRT1
Relative transcript level
(normalized by β-actin)
HIF-1α
SIRT1
β-Actin
RT-PCR
Hypoxia (24 h)
- + - +

## Slide 4
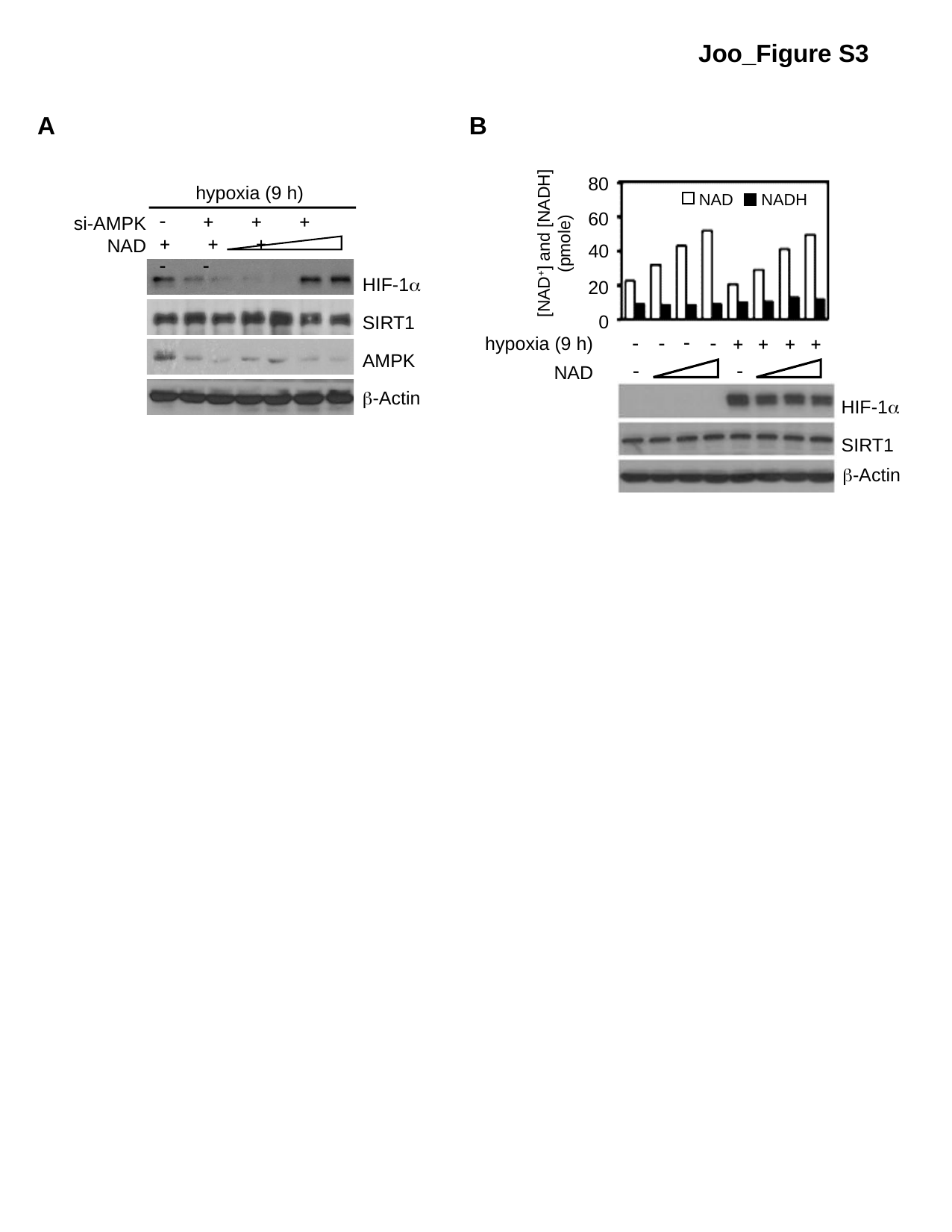

Joo_Figure S3
A
B
80
hypoxia (9 h)
NAD
NADH
60
- + + + + + +
- -
si-AMPK
NAD
[NAD+] and [NADH]
(pmole)
40
HIF-1α
SIRT1
AMPK
β-Actin
20
0
-
hypoxia (9 h)
-
-
-
+
+
+
+
-
-
NAD
HIF-1α
SIRT1
β-Actin

## Slide 5
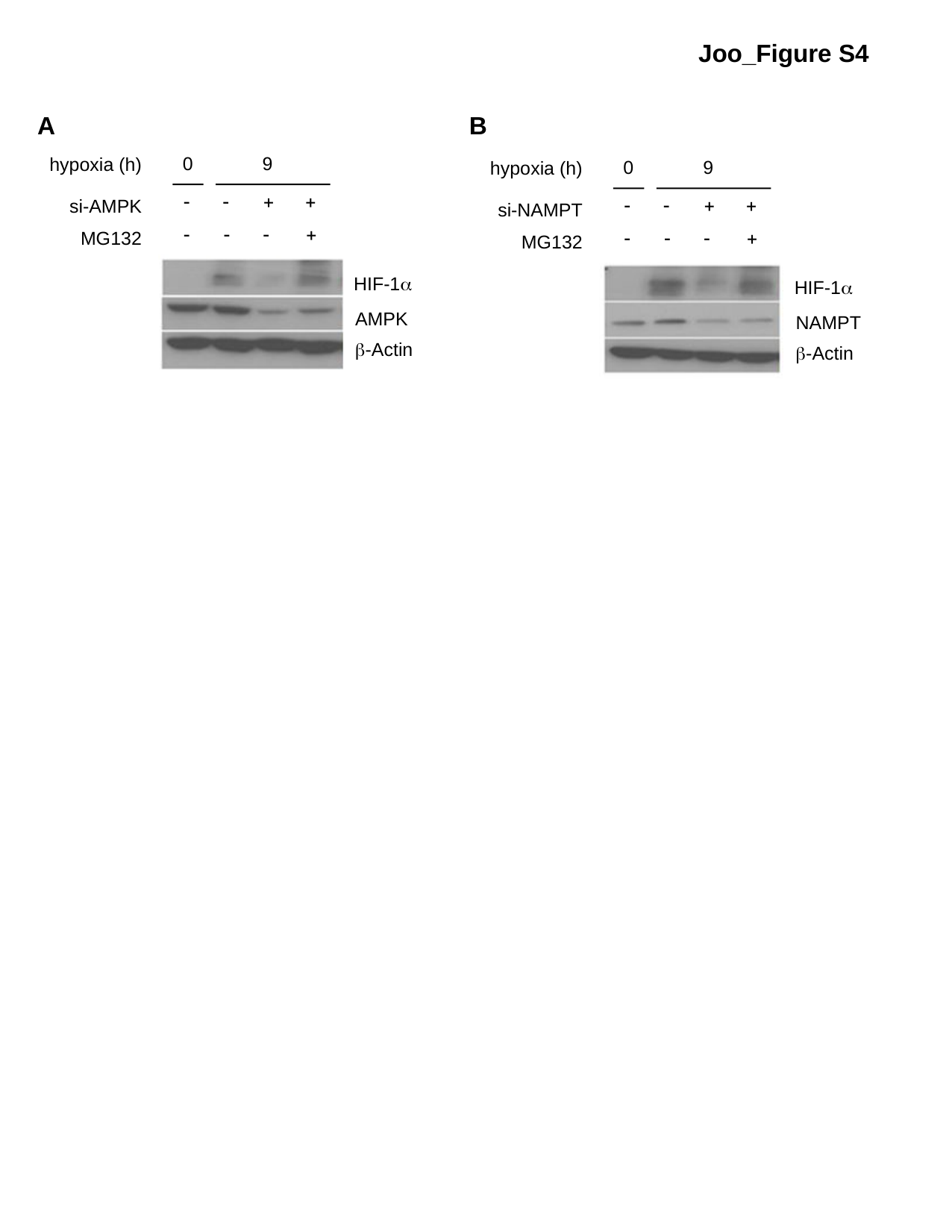

Joo_Figure S4
A
B
0
9
hypoxia (h)
0
9
hypoxia (h)
-
-
+
+
-
-
+
+
si-AMPK
si-NAMPT
-
-
-
+
MG132
-
-
-
+
MG132
HIF-1α
HIF-1α
AMPK
NAMPT
β-Actin
β-Actin

## Slide 6
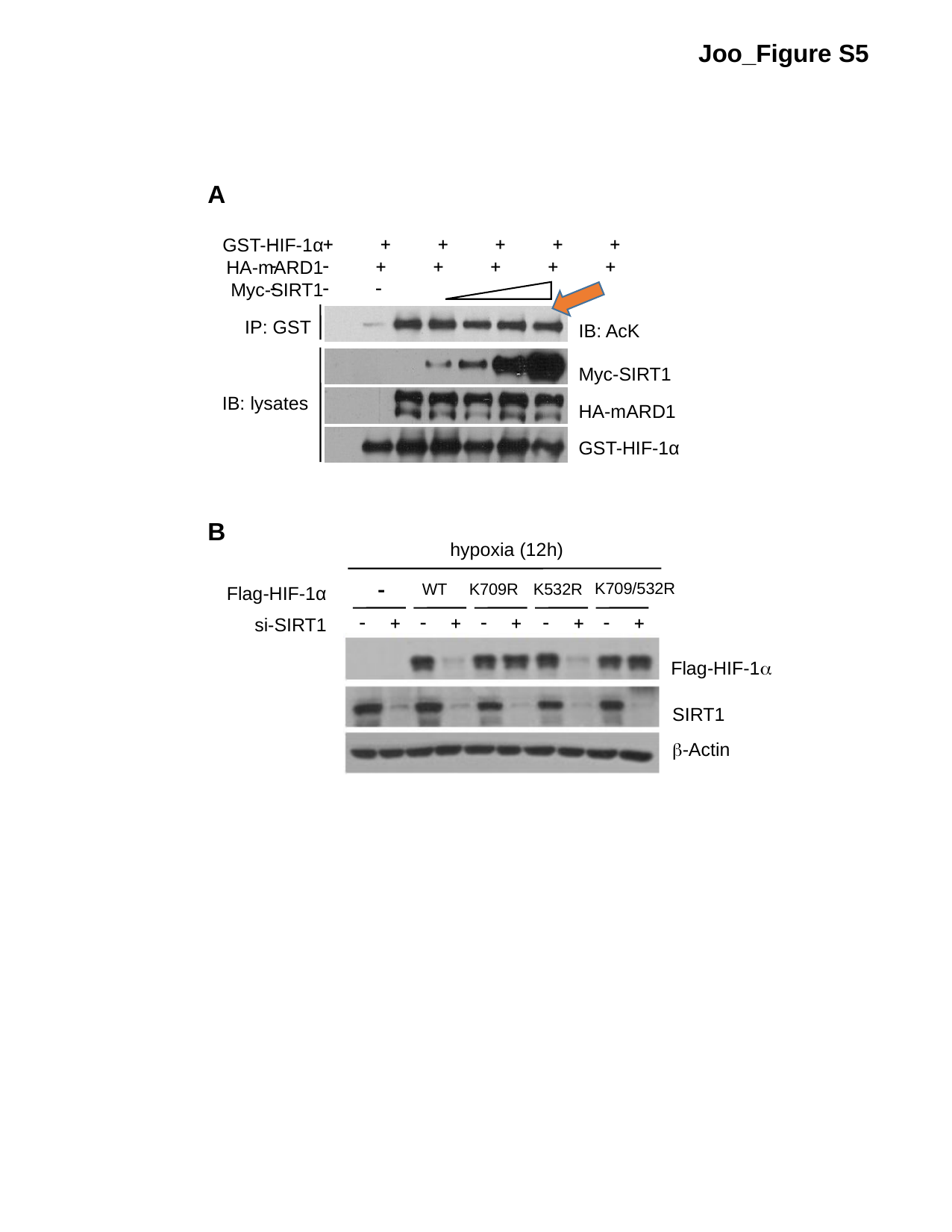

Joo_Figure S5
A
- + + + + + +
- - + + + + +
- - -
GST-HIF-1α
HA-mARD1
Myc-SIRT1
IB: AcK
Myc-SIRT1
HA-mARD1
GST-HIF-1α
IP: GST
IB: lysates
B
hypoxia (12h)
Flag-HIF-1α
-
K709/532R
WT
K709R
K532R
-
+
-
+
-
+
-
+
-
+
si-SIRT1
Flag-HIF-1α
SIRT1
β-Actin

## Slide 7
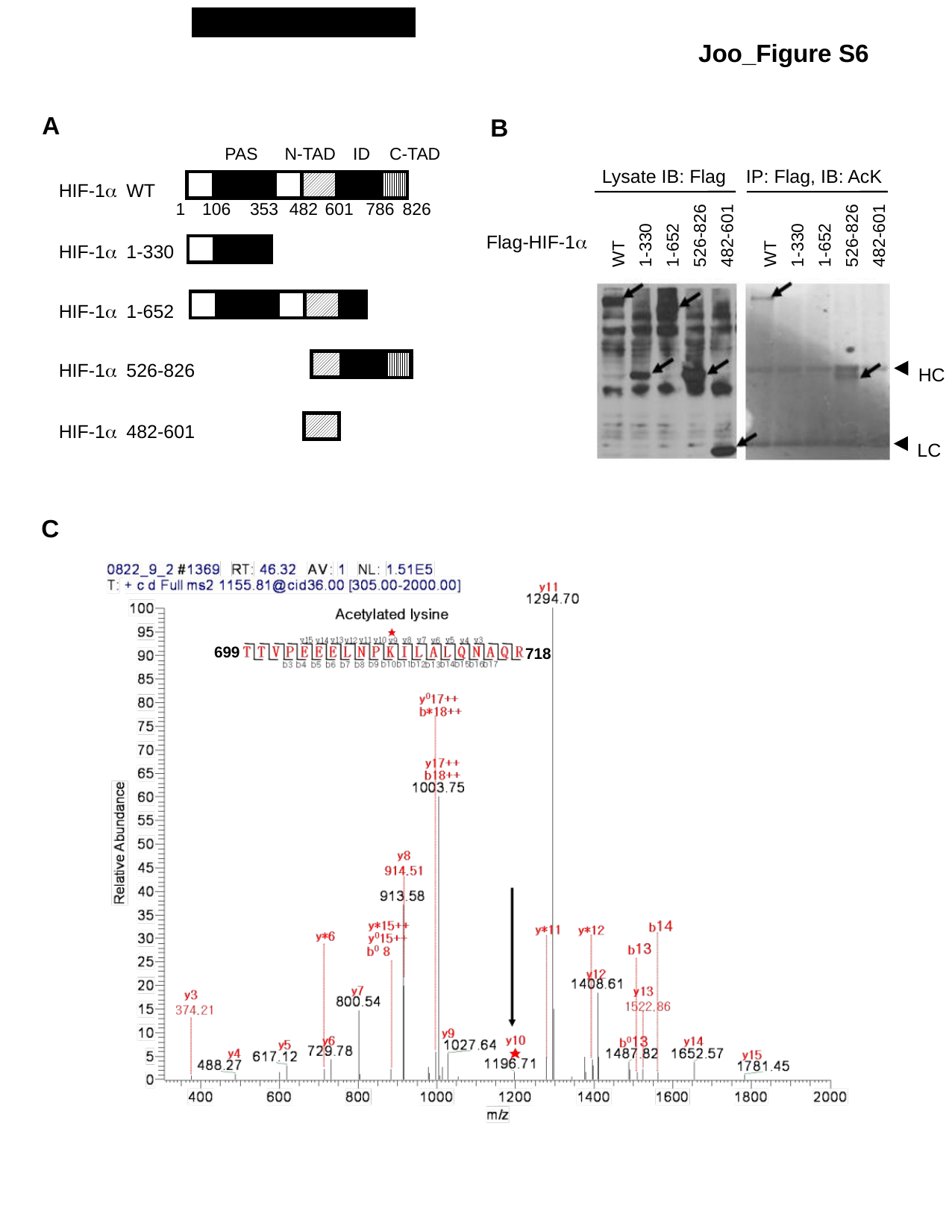

Joo_Figure S6
A
B
PAS
N-TAD
ID
C-TAD
Lysate IB: Flag
IP: Flag, IB: AcK
HIF-1α WT
1
106
353
482
601
786
826
526-826
482-601
526-826
482-601
Flag-HIF-1α
HIF-1α 1-330
1-330
1-652
1-330
1-652
WT
WT
HIF-1α 1-652
HIF-1α 526-826
HC
HIF-1α 482-601
LC
C
699
718

## Slide 8
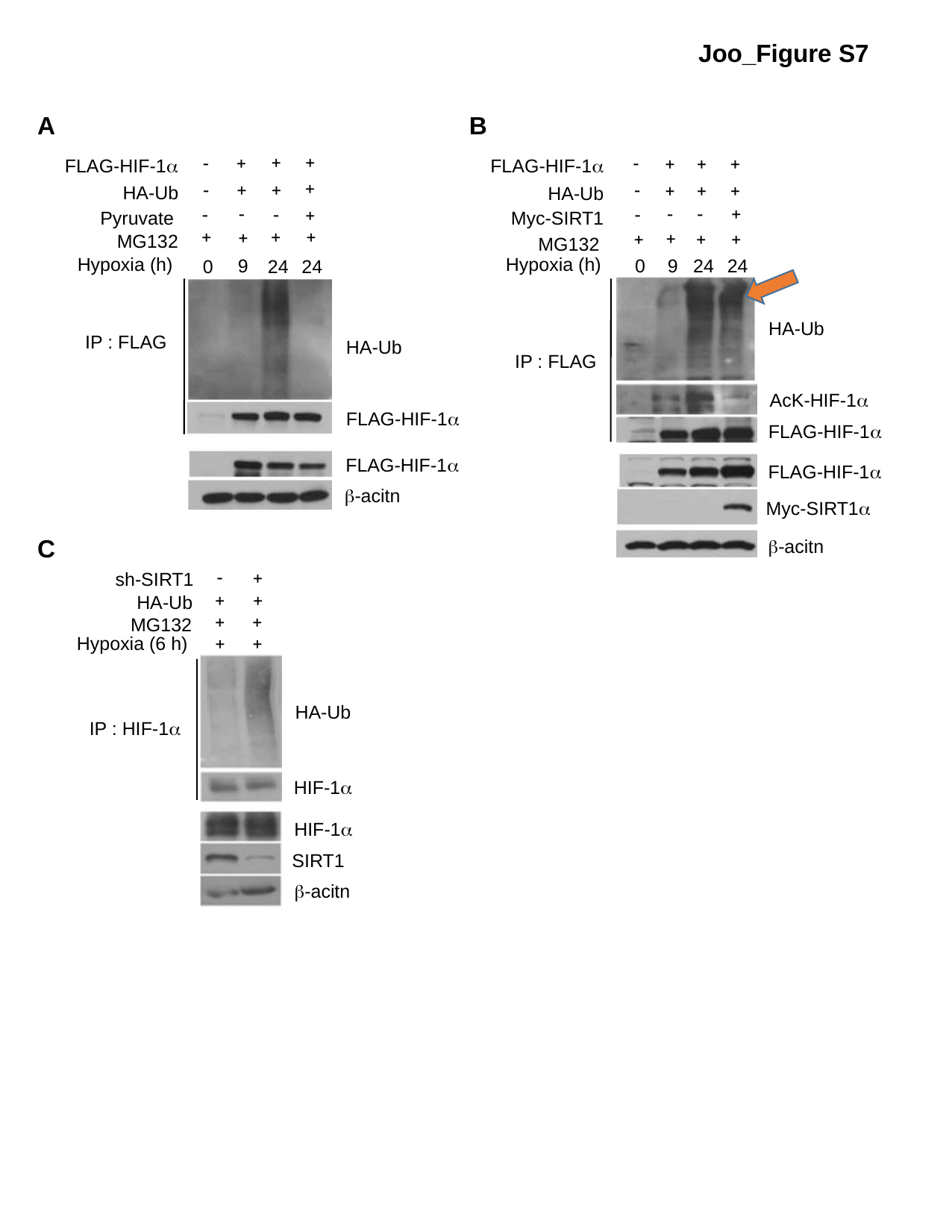

Joo_Figure S7
A
B
+
+
+
-
FLAG-HIF-1a
+
+
+
-
HA-Ub
-
-
-
+
Pyruvate
+
+
+
+
MG132
Hypoxia (h)
9
0
24
24
IP : FLAG
HA-Ub
FLAG-HIF-1a
FLAG-HIF-1a
b-acitn
-
+
+
+
FLAG-HIF-1a
+
-
+
+
HA-Ub
+
-
-
-
Myc-SIRT1
+
+
+
+
MG132
Hypoxia (h)
0
24
24
9
HA-Ub
IP : FLAG
AcK-HIF-1a
FLAG-HIF-1a
FLAG-HIF-1a
Myc-SIRT1a
b-acitn
C
-
+
sh-SIRT1
+
+
HA-Ub
+
+
MG132
Hypoxia (6 h)
+
+
HA-Ub
IP : HIF-1a
HIF-1a
HIF-1a
SIRT1
b-acitn

## Slide 9
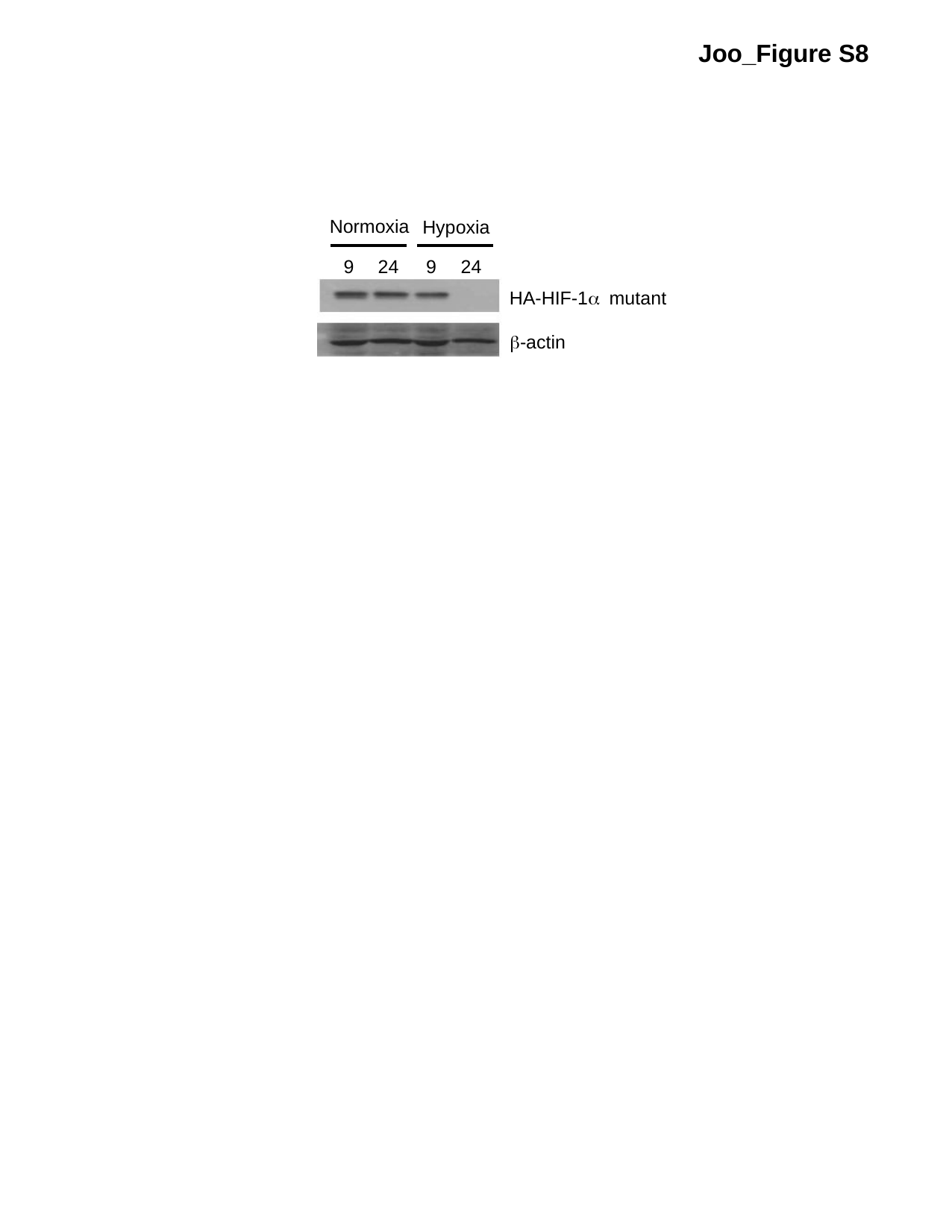

Joo_Figure S8
Normoxia
Hypoxia
9
9
24
24
HA-HIF-1a mutant
b-actin

## Slide 10
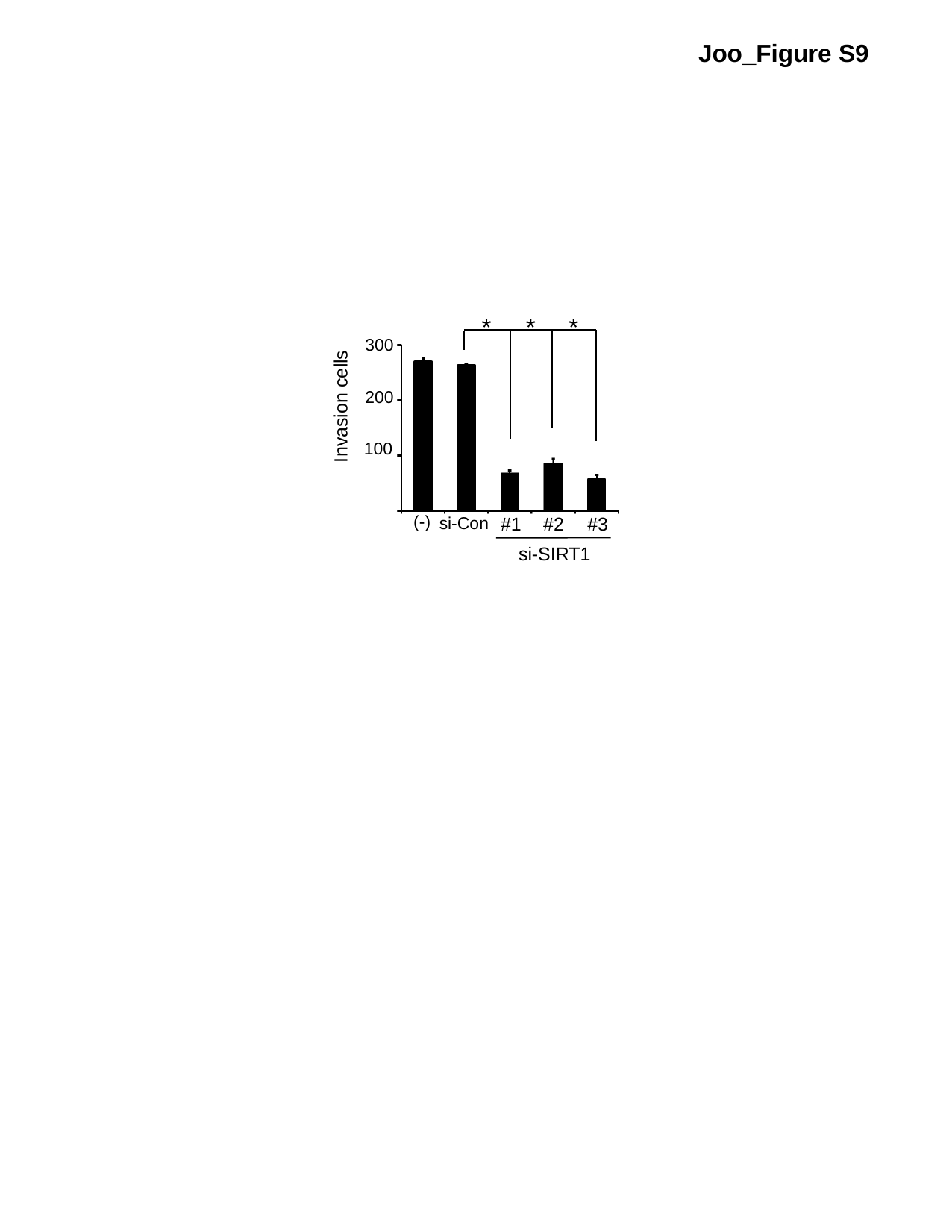

Joo_Figure S9
*
*
*
300
200
Invasion cells
100
(-)
si-Con
#1
#2
#3
si-SIRT1
