# Supplementary material for: NADH elevation during chronic hypoxia leads to VHL-mediated HIF-1α degradation via SIRT1 inhibition
Source: Cell Biosci. 2023 Sep 30;13:182. doi: 10.1186/s13578-023-01130-3 (PMC10543270; doi:10.1186/s13578-023-01130-3)
Supplement: Supplementary file 2 — Supplementary Material 2 [file 13578_2023_1130_MOESM2_ESM.docx]

**Supplementary Figure Legends**

**Figure S1. Chronic decay of HIF-1α during prolonged hypoxia is reversed by pyruvate.**

**(A)** HIF-1α protein levels were measured in medium with and without pyruvate during prolonged hypoxia. **(B)** Pyruvate-mediated recovery of HIF-1α during prolonged hypoxia was measured in HeLa cells following pyruvate treatment. (C) Association of SIRT1 with pyruvate-mediated recovery of HIF-1α was also validated in HeLa cells treateded with SIRT1 depletion using three different types of siRNA (#1, #2, and #3). (D) SIRT1-dependency of HIF-1α rescue was further explored in three established HeLa cell clones (#1, #7, and #9) featuring stable incorporation of shRNA directed toward SIRT1, and compared with that in wild-type HeLa and HeLa cells with control shRNA. Cells were incubated under hypoxic conditions for 24 h with addition of 1 mM pyruvate 6 h before harvesting.

**Figure S2. HIF-1α degradation is dependent on the proteasome pathway. (A)** HIF-1α protein levels were measured by MG132 treatment under acute and chronic hypoxic conditions. (B) HIF-1α degradation was inhibited by MG132 in HeLa cells treated with either SIRT1 depletion and pyruvate.(**C**) The amounts of *SIRT1* and *HIF-1α* mRNAs between HeLa cells before and 24 h after commencement of hypoxia were quantified by real time RT-PCR. The relative levels of *SIRT1* and *HIF-1α* mRNAs were determined by subtracting the threshold cycle of *β-Actin* from that of *SIRT1* and *HIF-1α*, respectively. (**D**) The amounts of *SIRT1* and *HIF-1α* mRNAs were measured by RT-PCR in the presence of pyruvate or NADH, or depletion of SIRT1.

**Figure S3. The NAD^+^ level under normoxic conditions is sufficient for HIF-1α stabilization.** (A) Under hypoxic conditions 9 h after commencement of hypoxia, the effect of exogenously added NAD^+^ on HIF-1α levels was evaluated in HeLa cells with (A) and without (B) AMPK depletion using siRNA. (B). The increasing concentrations of NAD^+^ (0.01, 0.03, 0.1, 0.3, or 1.0 mM in A, and 0.01, 0.1, or 1.0 mM in B were incubated before harvesting.

**Figure S4. Depletion of AMPK and NAMPT pathways destabilizes HIF-1α by proteasome mediated degardation.** (A,B) HIF-1α protein levels in the presence or absence of MG132 treatment were determined under NAD^+^-limited conditions achieved via depletion of AMPK (A) or NAMPT (B).

**Figure S5. Lys709 of HIF-1α is a deacetylation site mediated by SIRT1.** 293T cells were transiently cotransfected with 2 μg of each of GST-tagged HIF-1α, HA-tagged mouse ARD1 (mARD1), and/or increasing concentrations (0.5, 1, 3, and 6 μg) of Myc-tagged SIRT1. The acetylation levels of HIF-1α immunoprecipitated using antibodies against GST. (B) Genes encoding Flag-tagged forms of HIF-1α, including both wild-type (WT) and mutant (K709R, K532R, and K709R/K532R) proteins, were transiently transfected into HT1080 cells expressing SIRT1- or control-siRNA. HIF-1α levels were assessed using antibodies to the Flag tag.

**Figure S6. Acetylation region of and site in HIF-1α.** (A) Schematic domain map of HIF-1α fragments used for identification of the acetylation region. The fragments employed were the entire HIF-1α molecule, fragment aa 1-330, fragment aa 1-652, fragment aa 526-826, and fragment aa 482-601, of HIF-1α. (B) Acetylation region of HIF-1α. 293T cells were transiently cotransfected with Flag-tagged forms of each fragment together with an HA-tagged form of p300. Arrows indicate the exogenously expressed HIF-1α protein probed with an anti-Flag antibody (left panel) and the corresponding acetylated regions probed with an anti-acetyl K antibody (right panel). Immunoprecipitated HIF-1α was prepared with an antibody against Flag. (C) Acetylation site of HIF-1α as identified by MALDI-TOF. To determine the acetylation site, 293T cells were cotransfected with HIF-1α and p300, or HIF-1α and SIRT1 and then exposed to hypoxia. Modified HIF-1α was purified by immunoprecipitation. LC-MS/MS analysis was performed after a trypsin cleavage of the HIF-1α. The acetylated lysine 709 in the peptide fragment corresponding to aa 699-718 of HIF-1α was observed only in the sample cotransfected with HIF-1α and p300.

**Figure S7.** The extent of HIF-1α acetylation and HIF-1α interaction with Ubqutin is increased du**ring chronic hypoxia via SIRT1.**(A,B) The interaction of HIF-1α with ubqutin following presence pyruvation or SIRT1 overexpression under chronic hypoxia. HeLa cells transfected with FLAG-tagged HIF-1α (HA-VHL) and HA-tagged ubiquitin (HA-Ub) were exposed to hypoxic conditions in the presence of pyruvate (A) or in the presence of SIRT1- or control-shRNA (B). (C) The interaction of HIF-1α and ubqutin in SIRT1-deficient cells under acute hypoxia. SIRT1-deficient cells transfected with HA-Ub were exprosed to hypoxic condition for 9h.

**Figure S8. Chronic HIF-1α degradation is independent on the protein hydroxylation.** HeLa cells were transiently transfected with the plasmids encoding HA-tagged proline mutant P402A/P564A (Mutant). The levels of the mutant HIF-1α was determined under hypoxia and normoxia .

**Figure S9. Deplition of SIRT1 attenuates invasive of cancer cells under hypoxic conditions.** (A) Failure of invaive of cancer cells during 9 h of hypoxic exposure of HeLa cells, after SIRT1 depletion using three different types of siRNA (#1, #2, and #3) targeting SIRT1; the siRNA molecules bind to different regions on SIRT1 mRNA.
