# Supplementary material for: NADH elevation during chronic hypoxia leads to VHL-mediated HIF-1α degradation via SIRT1 inhibition
Source: Cell Biosci. 2023 Sep 30;13:182. doi: 10.1186/s13578-023-01130-3 (PMC10543270; doi:10.1186/s13578-023-01130-3)
Supplement: Supplementary file 3 — Supplementary Material 3 [file 13578_2023_1130_MOESM3_ESM.docx]

**Supplementary Tables**

**Table S1. siRNAs employed for gene silencing.**

| **Gene** | **Strand** | **Primer sequences** |
| --- | --- | --- |
| si-SIRT1  #1 | S | 5'-ACU UUG CUG UAA CCC UGU A-3' |
|  | AS | 5'-TAC AGG GUU ACA GCA AAG U-3' |
| si-SIRT1  #2 | S | 5'-5'-AGA GUU GCC ACC CAC ACC U-3' |
|  | AS | 5'-AGG UGU GGG UGG CAA CUC U-3' |
| si-SRT1  #3 | S | 5'-AAC CUUUGC CUC AUC UGC AUU-3' |
|  | AS | 5'-AAU GCA GAU GAG GCA AAG GUU-3' |
| sh-SIRT1 | S | 5'- cacca CAC CAG ATT CTT CAG TGA TTG TCA tctc TGA CAA TCA CTG AAG AAT CTG GTG G-3' |
|  | AS | 5'-aaaa CCA CCA GAT TCT TCA GTG ATT GTC A gaga TGA CAA TCA CTG AAG AAT CTG GTG G-3' |

**Table S2. PCR primers used for quantitative RT-PCR.**

| **Gene** | **Strand** | **Primer sequences** |
| --- | --- | --- |
| *HIF-1α* | S | 5'-CTC AAA GTC GGA CAG CCT CA-3' |
|  | AS | 5'-CCC TGC AGT AGG TTT CTG CT-3' |
| *SIRT1* | S | 5'-CAA ACT TTG CTG TAA CCC TGT-3' |
|  | AS | 5'-CAG CCA CTG AAG TTC TTT CAT-3' |
| *β-Actin* | S | 5'-AAG GAT TCC TAT GTC GGC-3' |
|  | AS | 5'-CAT CTC TTG CTC GAA GTC-3' |
|  |  |  |
|  |  |  |
